# Influence of Hydroxyl Group Position and Temperature on Thermophysical Properties of Tetraalkylammonium Hydroxide Ionic Liquids with Alcohols

**DOI:** 10.1371/journal.pone.0086530

**Published:** 2014-01-29

**Authors:** Pankaj Attri, Ku Youn. Baik, Pannuru Venkatesu, In Tae Kim, Eun Ha Choi

**Affiliations:** 1 Plasma Bioscience Research Center/Department of Electrical and Biological Physics, Kwangwoon University, Seoul, Korea; 2 Department of Chemistry, University of Delhi, Delhi, India; 3 Department of Chemistry, Kwangwoon University, Seoul, Korea; Jacobs University Bremen, Germany

## Abstract

In this work, we have explored the thermophysical properties of tetraalkylammonium hydroxide ionic liquids (ILs) such as tetrapropylammonium hydroxide (TPAH) and tetrabutylammonium hydroxide (TBAH) with isomers of butanol (1-butanol, 2-butanol and 2-methyl-2-propanol) within the temperature range 293.15–313.15 K, with interval of 5 K and over the varied concentration range of ILs. The molecular interactions between ILs and butanol isomers are essential for understanding the function of ILs in related measures and excess functions are sensitive probe for the molecular interactions. Therefore, we calculated the excess molar volume (*V^E^*) and the deviation in isentropic compressibility (Δ*κ_s_*) using the experimental values such as densities (*ρ*) and ultrasonic sound velocities (*u*) that are measured over the whole compositions range at five different temperatures (293.15, 298.15, 303.15, 308.15 and 313.15 K) and atmospheric pressure. These excess functions were adequately correlated by using the Redlich–Kister polynomial equation. It was observed that for all studied systems, the *V^E^* and Δ*κ_s_* values are negative for the whole composition range at 293.15 K. And, the excess function follows the sequence: 2-butanol>1-butanol>2-methyl-2-propanol, which reveals that (primary or secondary or tertiary) position of hydroxyl group influence the magnitude of interactions with ILs. The negative values of excess functions are contributions from the ion-dipole interaction, hydrogen bonding and packing efficiency between the ILs and butanol isomers. Hence, the position of hydroxyl group plays an important role in the interactions with ILs. The hydrogen bonding features between ILs and alcohols were analysed using molecular modelling program by using HyperChem 7.

## Introduction

Till date numerous research groups have focused their work on the study of fascinating physical properties of ionic liquids (ILs), due to their wide variety of applications in industries and applied chemistry [1]–[16]. For the applications in chemical and industrial processes, the knowledge of the thermophysical properties of IL is essential, as they represent the basis for the chemical and biological process [17]–[28]. Many of these studies have led to new possible applications for ILs [18]–[21]. Knowledge of structure and properties of ILs is essential for the understanding of their molecular interactions in the binary mixtures [4]–[6], [8]–[16]. Nevertheless, in order to transfer the ILs from laboratory to industry, designing future processes and equipment involving these ionic compounds, an accurate knowledge about their physical properties, either for pure ILs or mixed with other solvents, is crucial. Therefore, a deep knowledge of thermophysical properties of ILs and their liquid mixtures are essentially required for scientific community. Apparently, the physicochemical properties of ILs are quite sensitive toward the structure and nature of cations and anions [7]–[11]. The variations in thermophysical properties of ILs, such as density (*ρ*) and speed of sound (*u*) are observed to be very sensitive to the change in ion, mainly due to the microscopic level interactions between solvent molecules [12]–[16].

Binary mixtures of ILs with other solvents can also improve the thermodynamic and transport properties of working fluids as well as the efficiency of the chemical equipments such as batteries, photoelectrical cells, and other electrochemical apparatus. The use of the binary mixtures of ILs with polar compounds such as alcohols allows the change and control of the properties of the mixtures to suit a given situation [29].Thermodynamic properties of mixtures containing ILs and alcohols are important for both the design of many technological processes and an understanding of the solute–solvent interactions in the mixtures. These properties are required in the development of models for process design, energy efficiency, and in the evaluation of possible environmental impacts [30]. Regarding the study of physical properties for binary mixtures of alcohol+ILs, a large number of works have been published in recent years [31]–[41], showing the interest of the scientific community for this field. While, still there is no experimental or theoretical results are available for the thermophysical properties between the tetraalkylammonium hydroxide and butanol isomers. Additionally, there is no study to show the interactions between the hydroxide anion of the IL and hydroxyl group of alcohols.

In this research to study these interaction, we explore and compare the measurements of two thermophysical properties such as *ρ* and *u* of binary mixtures involving 1-butanol, 2-butanol and 2-methyl-2-propanol with tetrapropylammonium hydroxide [(C_3_H_7_)_4_N][OH] (TPAH) and tetrabutylammonium hydroxide [(C_4_H_9_)_4_N][OH] (TBAH) ILs over a complete mole fraction range at various temperatures from 293.15 to 313.15 K, with interval of 5 K. Further, the excess molar volume (*V^E^*), and deviation in isentropic compressibilities (Δκ_s_) were calculated using experimental data. The resulting *V^E^* and Δκ_s_ values were found to be strongly dependent on the place of hydroxyl group attached in the chain and also on the interactions between the hydroxide anion of ILs and hydroxyl group of the alcohols. These deviations in physical parameters have been explained in terms of intermolecular interactions between alcohols and ILs. Additionally, the temperature also plays an important role in interaction studies. Moreover, the hydrogen bonding features between ILs and alcohols were carried out to get a deep insight into intermolecular interactions for the studied compounds. These studies were performed according to the semi-empirical calculations by using HyperChem 7.

## Materials and Methods

### Materials

1-butanol, 2-butanol and 2-methyl-2-propanol were obtained from Merck >99% of purity and stored over freshly activated 3 Å molecular sieves and were purified by the standard method described by Riddick et al [42]. A comparison is made for the pure alcohols in Table 1 between the experimental *ρ* and *u* values determined in the present study and those reported in the literature [31], [38], [41]–[50]. ILs were synthesized in laboratory and analysed using ^1^H-NMR, the preparation is given below.

**Table 1:** Specifications of pure components and comparison of experimental densities (ρ) and ultrasonic sound velocities (u) with the literature values for alcohols.

Solvent	ρ/(g.cm^−3^)			u/(m.s^−1^)		
	T/K	Exptl.	Lit.	T/K	Exptl.	Lit.
1-Butanol	293.15	0.80980	0.80960 [ref 49]	293.15	1257	1256 [ref 42]
			0.80977 [ref 38]			1257 [ref 44]
			0.80917 [ref 46]			
			0.8094 [ref 44]			
			0.8098 [ref 43]			
			0.8095 [ref 42]			
	298.15	0.80567	0.80571 [ref 31]	298.15	1240	1239 [ref 42]
			0.80598 [ref 38]			1240 [ref 44]
			0.80554 [ref 46]			
			0.8058 [ref 44]			
			0.8060 [ref 43]			
			0.8057 [ref 42]			
	303.15	0.80195	0.80208 [ref 38]	303.15	1222	1222 [ref 42]
			0.80190 [ref 46]			1223 [ref 44]
			0.8018 [ref 44]			
			0.8021 [ref 43]			
			0.8019 [ref 42]			
			0.80221 [ref 48]			
	308.15	0.79821	0.79819 [ref 38]	308.15	1207	1206 [ref 42]
			0.79825 [ref 46]			1206 [ref 44]
			0.7979 [ref 44]			
			0.7982 [ref 43]			
			0.7980 [ref 42]			
			0.79834 [ref 48]			
						
	313.15	0.79420	0.79436 [ref 38]	313.15	1190	1189 [ref 42]
			0.79460 [ref 46]			
			0.7943 [ref 43]			
			0.7941 [ref 42]			
			0.79046 [ref 48]			
						
2-Butanol	293.15	0.80709	0.8063 [ref 42]	293.15	1229	1230 [ref 42]
			0.80657 [ref 46]			
						
	298.15	0.80267	0.8022 [ref 42]	298.15	1211	1212 [ref 42]
			0.80228 [ref 46]			
						
	303.15	0.79876	0.7980 [ref 42]	303.15	1193	1194 [ref 42]
			0.79892 [ref 45]			
			0.79799 [ref 46]			
			0.79835 [ref 48]			
			0.7989 [ref 47]			
						
	308.15	0.79446	0.7937 [ref 42]	308.15	1175	1176 [ref 42]
			0.79372 [ref 46]			
			0.79405 [ref 48]			
	313.15	0.79007	0.7893 [ref 42]	313.15	1157	1158 [ref 42]
			0.78943 [ref 46]			
			0.78965 [ref 48]			
			0.7901 [ref 47]			
2-Methyl-2-Propanol	293.15	0.78576		293.15	1145	
	298.15	0.78080	0.7812 [ref 41]	298.15	1123	
	303.15	0.77531	0.7753 [ref 47]	303.15	1102	
			0.77616 [ref 45]			
	308.15	0.76481	0.77036 [ref 48]	308.15	1080	
	313.15	0.76507	0.7648 [ref 47]	313.15	1059	
			0.76507 [ref 48]			

### Synthesis of ILs

#### Synthesis of Tetrapropylammonium Hydroxide (TPAH)

The synthesis of this IL was carried out in a 250 mL round bottomed flask, which was immersed in a water-bath, fitted with a reflux condenser. Solid potassium hydroxide (40 mmol) was added to a solution of tetrapropylammonium bromide [(C_3_H_7_)_4_N][Br] (40 mmol) in dry methylene chloride (20 mL), and the mixture was stirred vigorously at room temperature for 10 h. The precipitated KBr was filtered off, and the filtrate was then evaporated to leave the crude [(C_3_H_7_)_4_N][OH] as a viscous liquid that was washed with ether (2×20 mL) and dried at 343.15 K for 5 h to obtain the pure IL. The sample was analyzed by Karl Fisher titration and revealed very low levels of water (below 70 ppm). The yield of TPAH was 82%. ^1^H NMR (DMSOd_6_): δ (ppm) 0.8 (t, 12H), 1.46 (m, 8H), 2.92 (t, 8H), 4.56 (s, OH). HRMS calculated for C_12_H_29_NO (M+ - OH) 203.36, found 203.25.

#### Synthesis of Tetrabutylammonium Hydroxide (TBAH)

A procedure similar to that above for [(C_3_H_7_)_4_N][OH] was followed with the exception of the use of [(C_4_H_9_)_4_N][Br] ([cation]) instead of [(C_3_H_7_)_4_N][Br]. The yield of TBAH was 82%. ^1^H NMR (DMSOd_6_): δ (ppm) 0.94 (t, 12H), 1.37 (m, 8H), 1.96 (m, 8H), 3.43 (t, 8H), 4.78 (s, OH). HRMS calculated for C_16_H_37_NO (M+ - OH) 259.47 found out to be 259.34.

### Experimental Procedure

#### Density (ρ) and speed of sound (u) measurements

The density (*ρ*) and speed of sound (*u*) measurements were performed with an Anton-Paar DSA 5000 with an accuracy of temperature of ±0.01 K. The uncertainties in the density and speed of sound measurements were ±0.00005 g cm^−3^ and 0.01 m s^−1^ respectively. Prior to measurements, the instrument was calibrated with deionized water and dry air as standards at 293.15 K.

The binary mixtures of butanol isomers and IL were prepared by mass using a high-precision analytical balance with an uncertainty of ±1×10^−4^ g. All of the samples were prepared immediately before the measurements to avoid variations in composition due to evaporation of the solution. Clear and air bubble free solutions were used to perform the *ρ* and *u* experiments at different temperatures. The detailed measurement procedures used were described in detail in our previous research papers [12]–[15].

#### Hydrogen Bonding through Simulation Program

The structures of ILs and alcohols were optimized based on molecular mechanics and semi-empirical calculations using the HyperChem 7 molecular visualization and simulation program [51]–[54]. Initial molecular geometry of butanol isomers and ILs were optimized with the PM3 semi-empirical calculations and single point calculations were carried out to determine the total energies. Now the optimized molecules, alcohols and IL were chosen and then placed on top of each other symmetrically (parallel) with a starting interplanar distance of 2.3 Å and the angle made by covalent bonds to the donor and acceptor atoms less than 120^0^ was fulfilled. Further, the geometries were optimized using geometry optimizations based on molecular mechanics (using the MM+force field) and PM3 semi-empirical calculations, the Polak-Ribiere routine with rms gradient of 0.01 as the termination condition was used. PM3 uses a set of parameters derived from a variety of experimental versus calculated molecular properties, as compared to other semiempirical methods, including the AM1 procedure [53]. Typically, nonbonded interactions are less repulsive in the PM3 procedure [54]. Hydrogen bonds were displayed using HyperChem “show hydrogen bonds” and “recompute hydrogen bond” options.

## Results and Discussion

**Figure 1:**
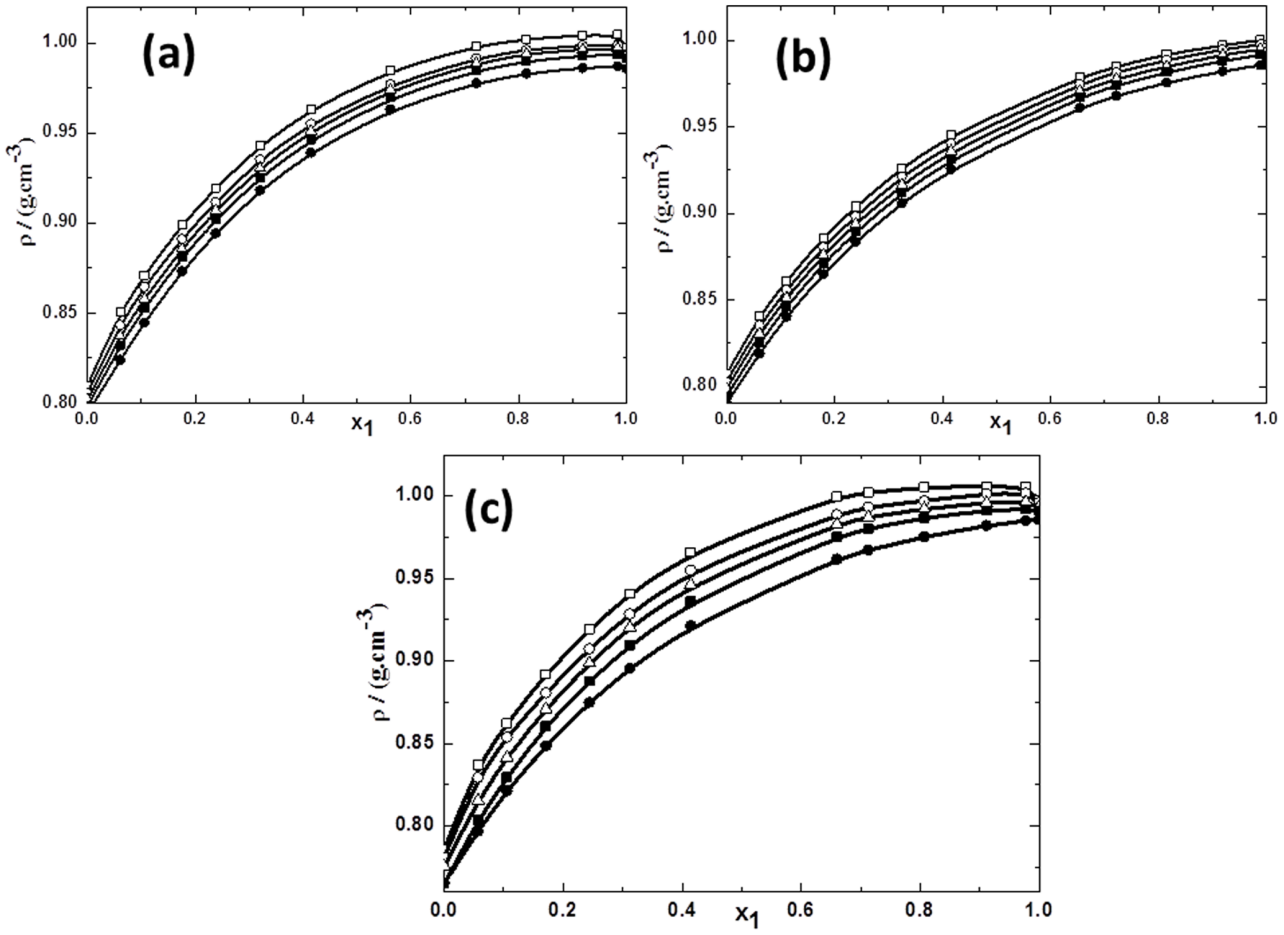
Densities for the mixtures of TPAH with alcohols vs mole fraction of IL *x*1 for (a) TPAH+1-butanol; (b) TPAH+2-butanol and (c) TPAH+2-methyl-2-propanol, 293.15 K (□),298.15 K (○), 303.15 K (▵), 308.15 K (▪),313.15 K (•) at various compositions and at atmospheric pressure. The solid line represents the smoothness of these data.

**Figure 2:**
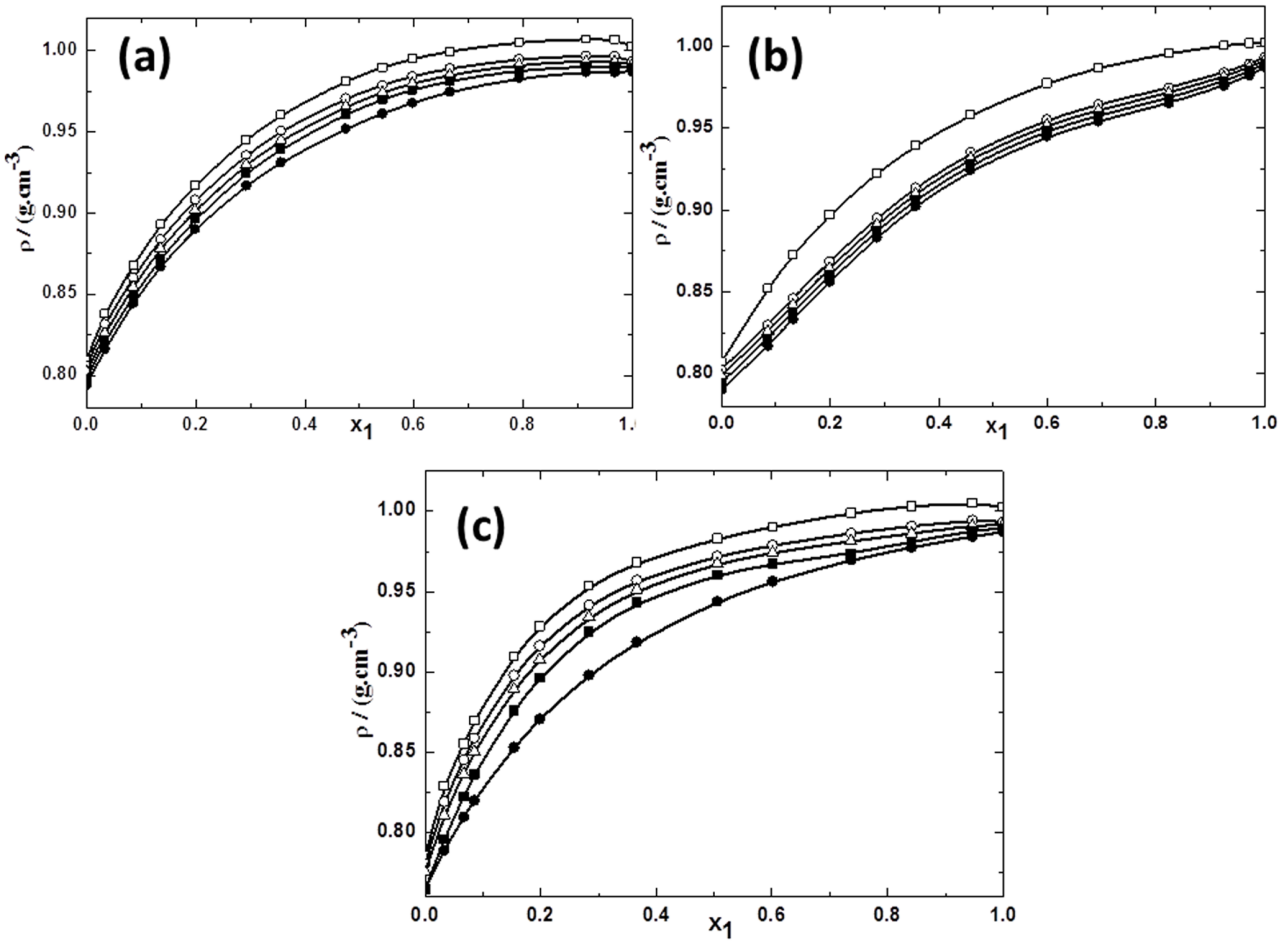
Densities for the mixtures of TBAH with alcohols vs mole fraction of IL *x*1 for (a) TBAH+1-butanol; (b) TBAH+2-butanol and (c) TBAH+2-methyl-2-propanol, 293.15 K (□), 298.15 K (○), 303.15 K (▵), 308.15 K (▪),313.15 K (•) at various compositions and at atmospheric pressure. The solid line represents the smoothness of these data.

**Figure 3:**
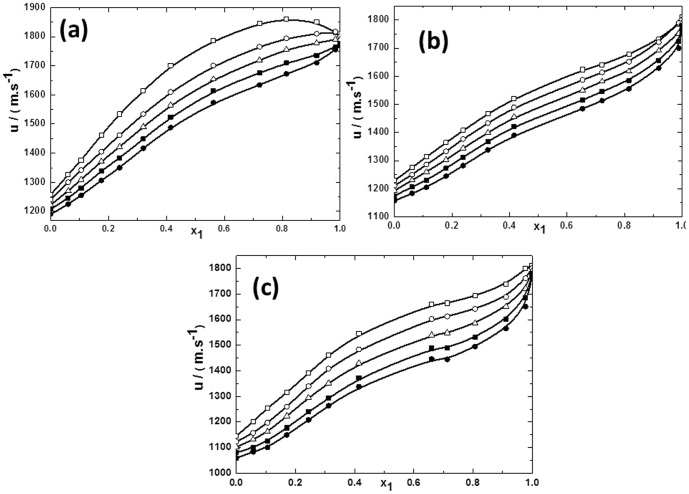
Ultrasonic sound velocity for the mixtures of TPAH with alcohols vs mole fraction of IL *x*1 for (a) TPAH+1-butanol; (b) TPAH+2-butanol and (c) TPAH+2-methyl-2-propanol, 293.15 K (□), 298.15 K (○), 303.15 K (▵), 308.15 K (▪),313.15 K (•) at various compositions and at atmospheric pressure. The solid line represents the smoothness of these data.

**Figure 4:**
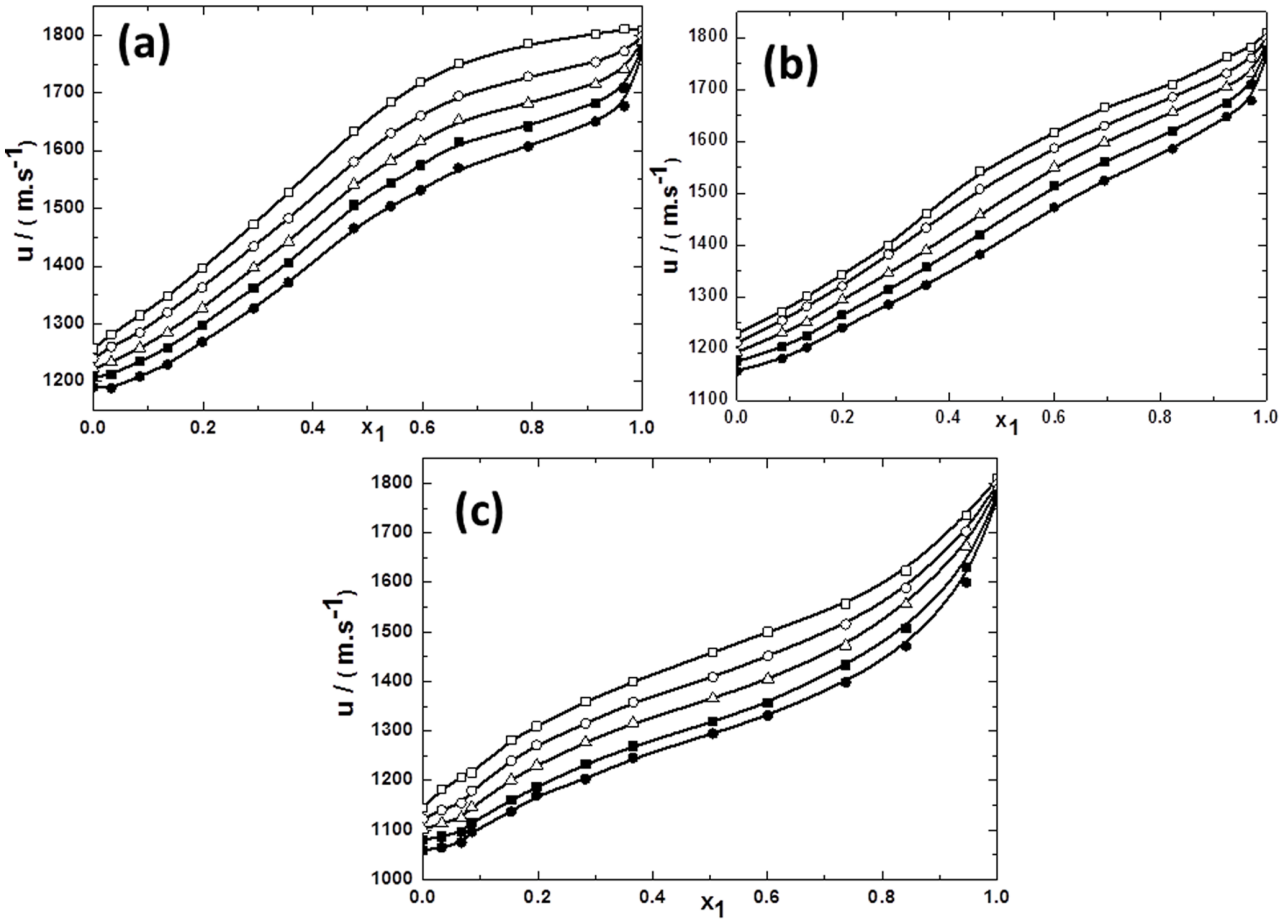
Ultrasonic sound velocity for the mixtures of TBAH with alcohols vs mole fraction of IL *x*1 for (a) TBAH+1-butanol; (b) TBAH+2-butanol and (c) TBAH+2-methyl-2-propanol, 293.15 K (□), 298.15 K (○), 303.15 K (▵), 308.15 K (▪),313.15 K (•) at various compositions and at atmospheric pressure. The solid line represents the smoothness of these data.

In order to have the better understanding of the molecular interactions between tetraalkylammonium hydroxide ILs with polar solvents such as 1-butanol, 2-butanol and 2-methyl-2-propanol, we have measured *ρ* and *u* properties over the whole composition range at various temperatures such as 293.15, 298.15, 303.15, 308.15 and 313.15 K under atmospheric pressure. The experimental *ρ* and *u* values of ILs with alcohols are presented as a function of IL concentration in Table S1 in File S1. Further, Figures 1–4 show the measured *ρ* and *u* values for the binary mixtures of different butanol isomers with both ILs (TPAH and TBAH) at all the studied temperatures. Figures 1 and 2 reveal that the variation of *ρ* values of TPAH or TBAH with 1-butanol, 2-butanol and 2-methyl-2-propanol, shows similar trends. It has been found that the *ρ* of the mixtures increased with the increasing concentrations of the ILs in alcohols. The effect of the ILs on the *ρ* in the alcohols has been examined at various temperatures. It has been observed that the *ρ* values decreased as temperature increased in the all systems. The results in Figure 1a clearly reveal that the *ρ* values of the TPAH+1-butanol mixture increase sharply up to *x*1≈0.8000 and later become almost constant at all the temperatures. While for TPAH+2-butanol, *ρ* values increase up to very rich IL concentration *x*1≈0.9900, as shown in Figure 1b. On the other hand, *ρ* values for TPAH+2-methyl-2-propanol increase sharply up to *x*1≈0.6400 and, no prominent changes have been observed afterwards. The increase in *ρ* values for TPAH+alcohols mixtures is possibly due to increase in the ion pair interactions between TPAH and alcohols. This shows that the density values of the TPAH+alcohol mixtures are not affected much due to change in the position of hydroxyl group in different isomers.

Whereas, in Figure 2a the *ρ* values for the TBAH+1-butanol mixtures increases sharply up to *x*1≈0.6200, later the increase is marginally very less at high concentration of IL region. Whereas, TBAH+2-butanol mixtures have similar trends as shown earlier by TPAH+2-butanol, *ρ* values increase up to very rich IL concentration *x*1≈0.9900, as depicted by Figure 2b. Further, TBAH+2-methyl-2-propanol mixture shows the increase in *ρ* values, while *ρ* doesn't increase sharply at mole fraction 0.5000–0.9900, which may be due to decrease in ion-pair interactions between TBAH and 2-methyl-2-propanol, as shown in Figure 2c. From Table S1 in File S1, we observed that the densities of investigated systems increase with increasing the length of alkyl chain in IL. It was found that *ρ* values to be higher in the TBAH+butanol isomers as compared to TPAH+butanol isomers at equimolar mixture. Whereas, according to early documented research articles the density decreases with increase in alkyl chain in a cation or anion [55], [56]. These discrepancies vary from IL to IL and solvent to solvent and also depend on the nature as well as structural arrangement of IL and solvent. Moreover, from close look on the Table S1 in File S1, we observed that with increase in temperature, *ρ* values of TBAH+butanol isomers decreases more as compared to TPAH+butanol isomers. This might be due to the assumption that the ion-pair interaction decreases more for high alkyl chain+butanol isomers as compared to lower alkyl chain+butanol isomers with the increase in temperature.

Ultrasonic sound velocities (*u*) prove to be an informative source regarding the properties of different solvents and their mixture. The values of *u* were found to decrease with an increase in temperature while *u* values increased with increasing in mole fraction of IL. As noted from Figures 3 and 4, there is a sharp increase of *u* in all ILs, except in the mixture of TPAH with 1-butanol at 293.15 K, in the mole fraction range from 0.8000 to 0.9900 of IL. Over this range, the *u* values decrease slightly for the mixtures of TPAH with 1-butanol at 293.15 K. Whereas, no change is observed in rest of the IL+butanol isomers at all investigated temperatures. This *u* value is significantly increased in IL-solvent interactions when the mole fraction of IL was increased. If we compare the TBAH+1-butanol to TPAH+1-butanol, it has been observed that the *u* values slightly decrease when the alkyl substituents size of cation increases. Whereas, the same trend was observed on comparing the *u* values of TBAH+2-methyl-2-propanol to TPAH+2-methyl-2-propanol. It has been found that the *u* values slightly decrease when the size of cation increases. While the *u* value for TPAH+2-butanol are lower than TBAH+2-butanol, which again reveal that *u* values slightly increase as the size of cation increases. Hence, our results lead to conclusion that interactions of ILs with alcohols, depends upon the position of the hydroxyl group.

**Figure 5:**
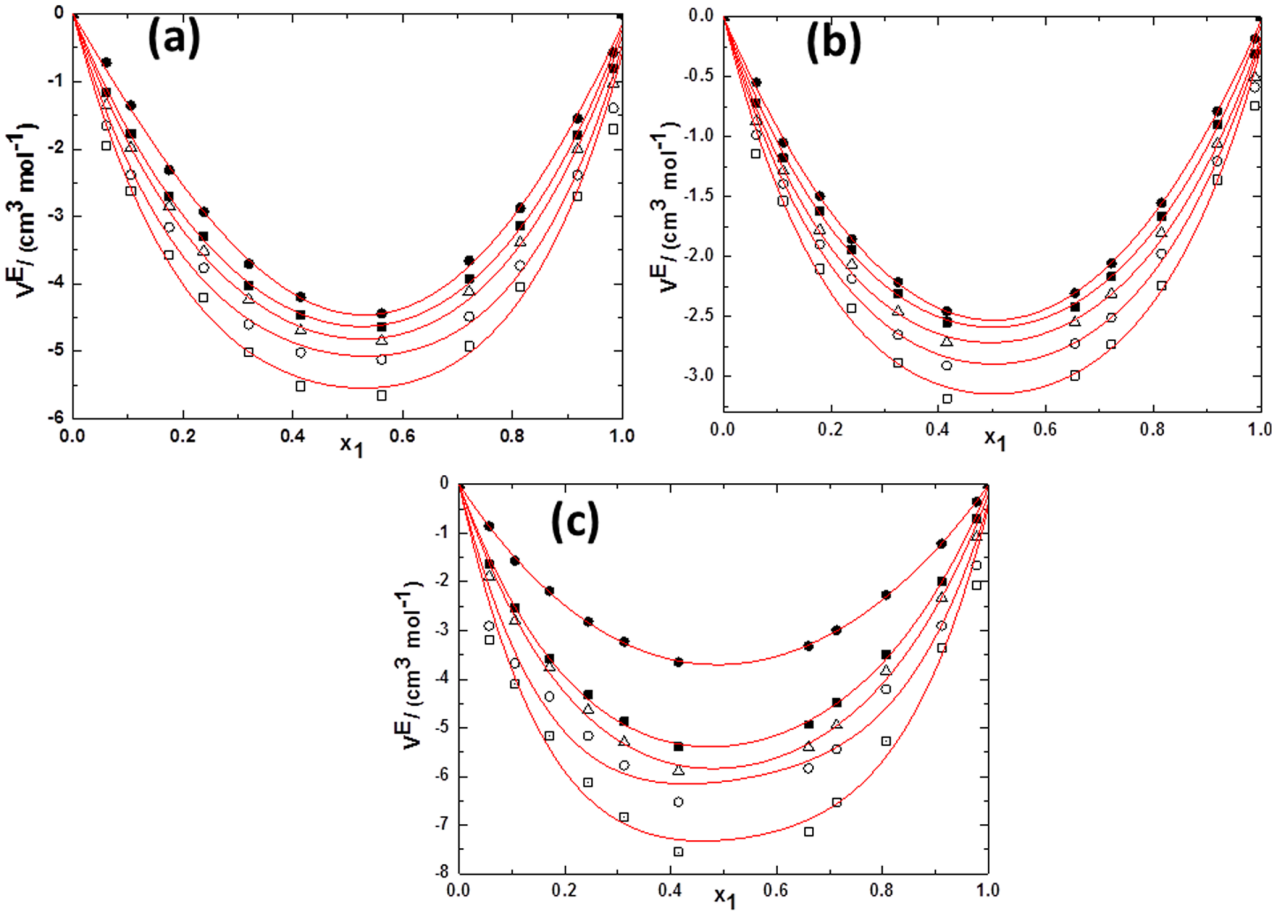
Excess molar volumes (*V^E^*) against the mole fraction of TPAH *x*1 for (a) TPAH+1-butanol; (b) TPAH+2-butanol and (c) TPAH+2-methyl-2-propanol, 293.15 K (□), 298.15 K (○), 303.15 K (▵), 308.15 K (▪),313.15 K (•) at various compositions and at atmospheric pressure. Solid lines correlated by the Redlich-Kister equation.

**Figure 6:**
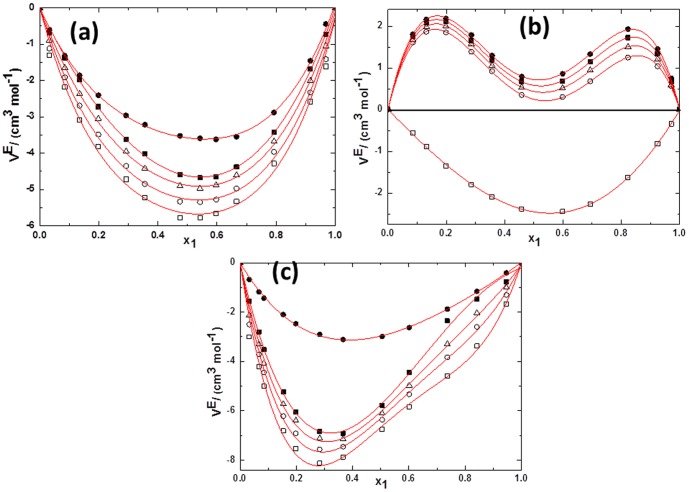
Excess molar volumes (*V^E^*) against the mole fraction of TBAH *x*1 for (a) TBAH+1-butanol; (b) TBAH+2-butanol and (c) TBAH+2-methyl-2-propanol, 293.15 K (□), 298.15 K (○), 303.15 K (▵), 308.15 K (▪),313.15 K (•) at various compositions and at atmospheric pressure. Solid lines correlated by the Redlich-Kister equation.

**Figure 7:**
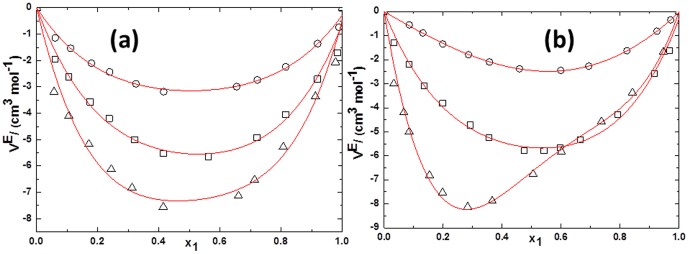
Excess molar volumes (*V^E^*) of ILs+alcohols at293.15 K for (a) TPAH+1-butanol(□), TPAH+2-butanol (○) and TPAH+2-methyl-2-propanol (▵); (b) TBAH+1-butanol(□), TBAH+2-butanol (○) and TBAH+2-methyl-2-propanol (▵) at atmospheric pressure. Solid lines correlated by the Redlich-Kister equation.

**Figure 8:**
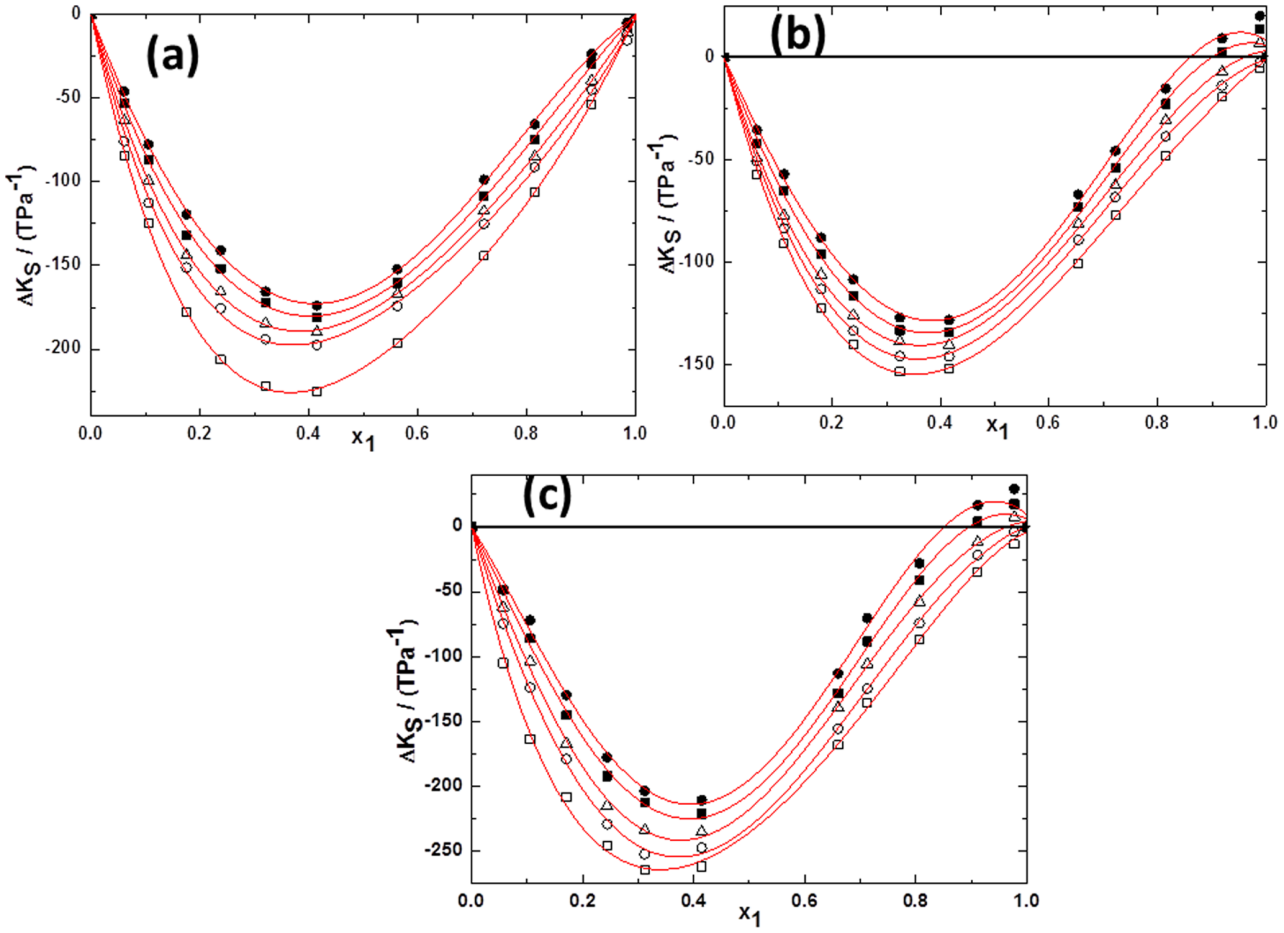
Deviation in isentropic compressibilities (Δ*κ_s_*) against the mole fraction of TPAH *x*1 for (a) TPAH+1-butanol; (b) TPAH+2-butanol and (c) TPAH+2-methyl-2-propanol, 293.15 K (□), 298.15 K (○), 303.15 K (▵), 308.15 K (▪),313.15 K (•) at various compositions and at atmospheric pressure. Solid lines correlated by the Redlich-Kister equation.

**Figure 9:**
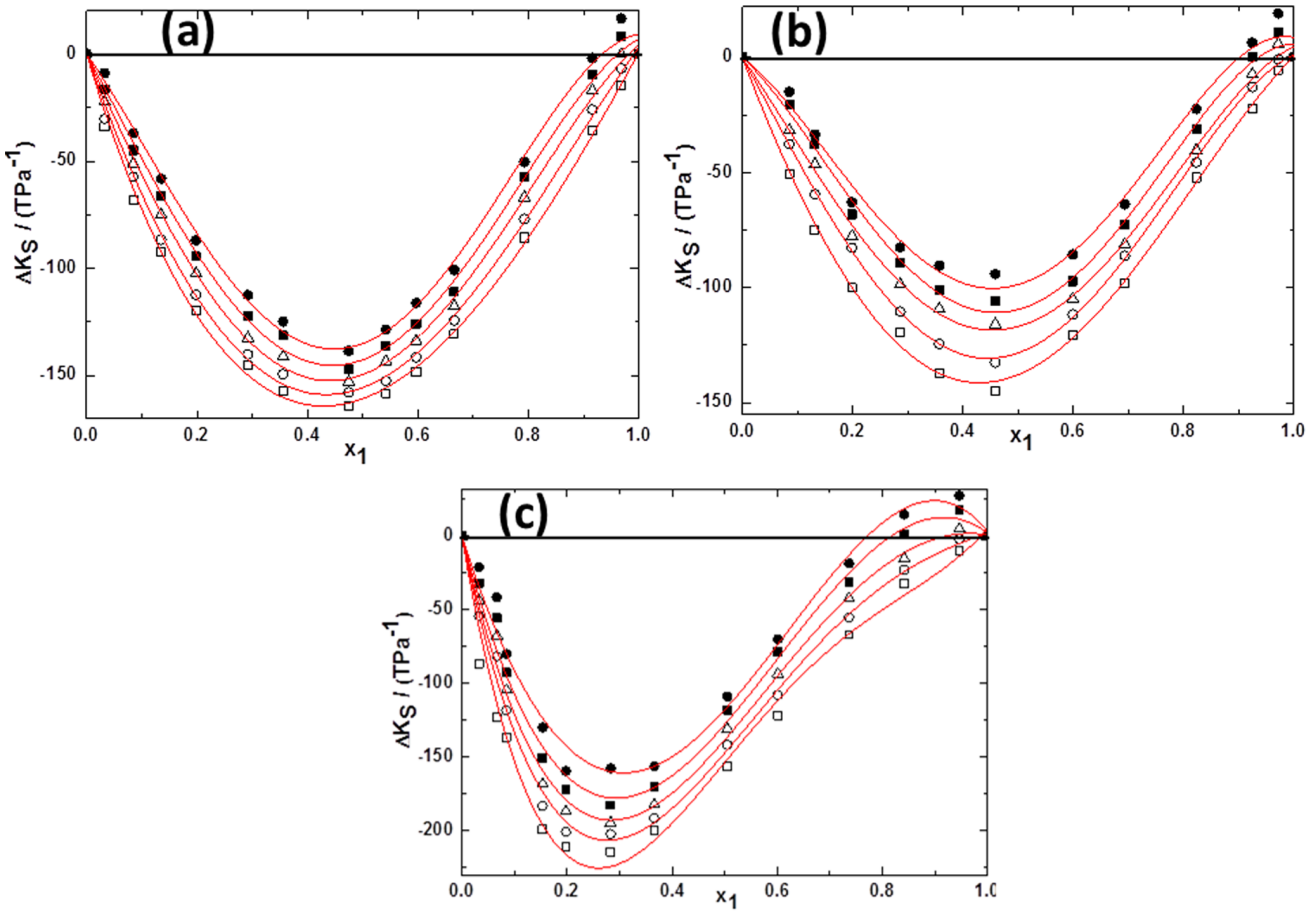
Deviation in isentropic compressibilities (Δ*κ_s_*)against the mole fraction of TBAH *x*1 for (a) (a) TBAH+1-butanol; (b) TBAH+2-butanol and (c) TBAH+2-methyl-2-propanol, 293.15 K (□), 298.15 K (○), 303.15 K (▵), 308.15 K (▪),313.15 K (•) at various compositions and at atmospheric pressure. Solid lines correlated by the Redlich-Kister equation.

**Figure 10:**
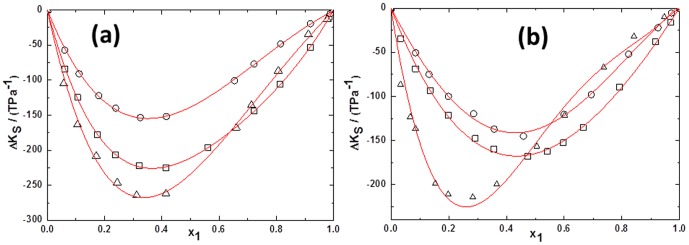
Deviation in isentropic compressibilities (Δ*κ_s_*) of ILs+alcohols*x*1 at293.15 K for (a) TPAH+1-butanol(□), TPAH+2-butanol (○)and TPAH+2-methyl-2-propanol(▵);(b) TBAH+1-butanol(□), TBAH+2-butanol (○) and TBAH+2-methyl-2-propanol(▵)at atmospheric pressure. Solid lines correlated by the Redlich-Kister equation.

Thermophysical properties of mixed solvents of ILs with butanol isomers can be tunable. The extent of deviation of liquid mixtures from ideal behavior is best expressed by excess functions. Excess molar volumes (*V^E^*) as well as ultrasonic studies are known to provide useful insights into solution structural effects and intermolecular interactions between component molecules. The extent of deviation of liquid mixtures from ideal behavior is best expressed by excess functions. Volumetric properties of binary mixtures of ILs with polar compounds are contributed to the clarification of the various intermolecular interactions existing between the different species found in solution. The excess volumes are determined from the density of pure compounds (*ρ*_1_ and *ρ*_2_) and mixture (*ρ*_m_) using a standard equation [15]. The ultrasonic studies have been adequately employed in understanding the nature of molecular interaction in solvent mixed systems. In the chemical industry, knowledge of the ultrasonic and its related properties of solutions are essential in the design involving chemical separation, heat transfer, mass transfer, and fluid flow. Isentropic compressibilities (*κ_s_*) of the binary mixtures were calculated using the relation from *ρ* and *u*. The composition dependence of the *V^E^* and Δ*κ_s_* properties represents the deviation from ideal behavior of the mixtures and provides an indication of the interactions between IL and alcohols. These properties were mathematically fitted by variable degree functions using the Redlich-Kister expression:
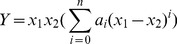
(1)

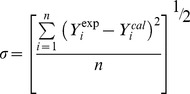
(2)Where *Y* refers to *V^E^* or Δ*κ_s_*. *ai* are adjustable parameters and can be obtained by least-squares analysis. Values of the fitted parameters are listed in Table S2 in File S1, along with the standard deviations of the fit. The values of *V^E^* and Δ*κ_s_* for the binary mixtures at various temperatures as function of ILs concentrations are included in Table S1 in File S1. Figures 5 to 10 display the experimental data for the binary mixtures, and the fitted curves, along with the excess properties of *V^E^* and Δ*κ_s_* for the butanol isomers with ILs as function of IL concentrations at different temperatures.

From Figure 5 one can note that the values of *V^E^* are negative for all TPAH+butanol isomers systems at all measured temperatures over whole composition range. We have observed that the excess molar volumes present a minimum at *x*1≈0.5634 for TPAH+1-butanol at all investigated temperatures, whereas we obtained that the *V^E^* values present a minimum at *x*1≈0.4143 for the TPAH+2-butanol system. Further, minimum *V^E^* values lie at *x*1≈0.4244 for TPAH+2-methyl-2-propanol system at all investigated temperatures. The minimum *V^E^* values could be due to hydrogen bonds between alcohols and TPAH IL. The decrease in the magnitude of the negative *V^E^* values with an increase in the IL composition can be attributed to the decrease of hydrogen bonding. In other words, due to increase in the concentration of the IL results in decrease of packing efficiency. Further, with increase in temperature the magnitude of the negative *V^E^* values decreases in all the TPAH+butanol isomers systems. This can again be due to decrease in magnitude of the hydrogen bonding with increase in the temperature.

It is interesting to note that the *V^E^* values in TPAH+2-methyl-2-propanol mixture show more negative values of *V^E^* at the alcohol-rich composition than the TPAH+1-butanol and TPAH+2-butanol mixtures at 293.15 K (Figure 7a), implying that in the TPAH+2-methyl-2-propanol, there are ion-dipole interactions and packing effects with 2-methyl-2-propanol which are stronger than those in the 2-butanol and 1-butanol solution at *x*1≈0.4200. A comparison between the negative deviation of *V^E^* of TPAH+2-methyl-2-propanol, TPAH+1-butanol and TPAH+2-butanol suggests that there is a difference of the hydroxyl position in the alkyl chain, leading to variation in the interactions between the alcohols and TPAH. Molecular interaction between TPAH and alcohols follows the following order at 293.15 K, 2-methyl-2-propanol>1-butanol>2-butanol.

Further, the negative *V^E^* values are observed for TBAH+butanol isomers at all measured temperatures over whole composition range, except 2-butanol at higher temperatures 293.15 to 313.15 K. The *V^E^* values for 1-butanol with TBAH are as represented in Figure 6a. And, we found that negative *V^E^* values are observed over the entire mole fraction range at all investigated temperatures. These negative *V^E^* values reveal that a more efficient packing or attractive interaction occurred between the TBAH and 1-butanol. 1-Butanol forms a hydrogen bond with the alkyl chain cation, while the interactions decrease at higher temperatures. The interactions between the 1-butanol molecules and the alkyl chain of TBAH are due to ion-dipole or hydrogen bonding interactions. This will reduce the interactions between the tetrabutylammonium cation and hydroxide anion in the IL, which contributes to the negative *V^E^* values. Furthermore, the observed positive *V^E^* values for TBAH+2-butanol at higher temperatures show that there exist no specific interactions between unlike molecules, as displayed in Figure 6b. The magnitude and sign of *V^E^* values are a reflection of the type of interaction staking place in the mixture, which are the result of different effects containing the loss of the dipole interaction from each other and the breakdown of the IL ion pair (positive *V^E^*). The interaction between the ion pair of ILs increases as compared to IL+2-butanol interactions, which leads to positive contribution at higher temperature over whole composition range. While, Figure 6c shows the negative *V^E^* values for TBAH+2-methyl-2-propanol at all measured temperatures over whole composition range. This might be due to the large difference between the molar volumes of the 2-methyl-2-propanol and TPAH implying that it is possibly due to the fact that the relatively small organic molecules fit into the interstices upon mixing. Therefore, the filling effect of organic molecular liquids in the interstices of ILs, and the ion-dipole interactions between organic molecular liquid and alkyl cation of ammonium ILs, all contribute to the negative values of *V^E^*.

Clearly, the observed negative *V^E^* values increases further with increasing the temperature in the entire mole fraction range for all IL systems. It is interesting to note that the *V^E^* values in the ILs+2-methyl-2-propanol mixture shows more negative values of *V^E^* than the IL+2-butanol and IL+1-butanol mixtures at 298.15 K over the alcohol rich concentration range (Figure 7), implying that in the 2-methyl-2-propanol there is strong ion-dipole interactions and packing effects with ILs as compared to 2-butanol and 1-butanol. The magnitude and sign of *V^E^* values are a reflection of the type of interactions taking place in the mixture, which reveals that *V^E^* values are more negative for TBAH (*V* ^E^ = −8.149 cm^3^.mol^−1^ at *x*_1_ = 0.2831 for TBAH+2-methyl-2-propanoland *V* ^E^ = −5.787 cm^3^.mol^−1^ at *x*_1_ = 0.5428 for TBAH+2-butanol) than TPAH (*V* ^E^ = −7.547 cm^3^.mol^−1^ at *x*_1_ = 0.4143 for TPAH+2-methyl-2-propanol and *V* ^E^ = −5.702 cm^3^.mol^−1^ at *x*_1_ = 0.5634 for TPAH+2-butanol) in all systems except in ILs+1-butanol systems. For TBAH+1-butanols (*V* ^E^ = −2.448 cm^3^.mol^−1^ at *x*_1_ = 0.5993) and *V* ^E^ = −3.175 cm^3^.mol^−1^ at *x*_1_ = 0.4139 for TPAH+1-butanol, hence the *V* ^E^ is more negative for the TPAH+1-butanol than TBAH+1-butanol due to steric hindrance created by the long chain cation of TBAH, that reduces the interaction magnitude between TBAH and 1-butanol. Interestingly, the hydrogen bonding between ILs and butanol isomers has predicted using semiempirical calculations with the help of Hyperchem 7, and those interactions are explicitly elucidated in Figures S1, S2, S3, S4, S5, S6.

Using semiempirical calculations for the hydrogen bonding between ILs and butanol isomers, displayed in Figures S1, S2, S3, S4, S5, S6, we calculated heat of formation of the complexes and compared the values with those of the ILs and butanol isomers (as displayed in Table 2). In all the cases, Δ*H_f_* of the complex resulting from hydrogen bonding was higher than the sum of Δ*H_f_*'s of butanol isomers and ILs. It is reasonable to assume that these differences (ΔΔ*H_f_*), calculated according to equation 3, represent the energies of the hydrogen bond. The energies of the hydrogen bonding can also obtained by using the total binding energies of butanol isomers [54] and ILs, presented in Table 2, instead of Δ*H_f_*'s for these calculations. The results in Table 2 indicate that the energy required for the formation of a weak hydrogen bond is less than required for the formation of a stronger hydrogen bond:
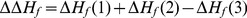
(3)Where Δ*H_f_* (1) is the heat of formation of the butanol isomers, Δ*H_f_* (2) the heat of formation of the ILs and Δ*H_f_* (3) the heat of formation of the complex (butanol isomers and ILs).

**Table 2:** Calculated binding energies (*E*), heats of formations (Δ*H_f_*), and estimated hydrogen bond energies (ΔΔ*H_f_*) (kcal/mol).

Solvent	*E*/(kcal/mol)	Δ*H_f_*/(kcal/mol)	ΔΔ*H_f_*/(kcal/mol)
TPAH	−3784.96	−50.76	
TBAH	−4907.84	−73.27	
1-Butanol	−1330.63	−66.49	
2-Butanol	−1330.86	−66.72	
2-Methyl-2-Propanol	−1333.75	−69.61	
TPAH+1-Butanol	−5117.47	−119.12	1.87
TPAH+2-Butanol	−5119.10	−121.62	4.14
TPAH+2-Methyl-2-Propanol	−5118.74	−121.01	0.64
TBAH+1-Butanol	−6238.88	−140.15	0.39
TBAH+2-Butanol	−6241.01	−142.28	2.29
TBAH+2-Methyl-2-Propanol	−6241.09	−142.96	0.08

A glance at the Figure S1, illustrated the hydrogen bonding between nitrogen group of TPAH IL with the “-OH” group of 1-buatnol. The binding energy of the TPAH is found to be −3784.96 kcal/mol and that of 1-butanol is found to be −1330.63 kcal/mol, but after the hydrogen bonding occur between the TPAH and 1-butanol, the binding energy of complete system comes out to be −5117.47 kcal/mol (Table 2). Hence, the estimated hydrogen bond energy of the above system is ≈1.87 kcal/mol, which could probably due to the interaction of TPAH with 1-butanol that leads to decrease in energy (less than sum of individual energy of TPAH and 1-butanol), and increase in the strength of hydrogen bonding. Similarly, the Figures S2 and S3, depict the possibility of hydrogen bonding between the nitrogen group of TPAH IL with hydroxyl group of 2-butanol and 2-methyl-2-propanol and the estimated hydrogen bond energies are ≈4.14 and ≈0.64 kcal/mol respectively (Table 2). This shows that the strength of hydrogen bonding is more for TPAH+2-butanol as compared to other butanol isomers. Further, the Figures S4 to S6, clearly again show the hydrogen bonding between the nitrogen group of TBAH with hydroxyl group of 1-butanol, 2-butanol and 2-methyl-2-propanol and now the hydrogen bond energies are ≈0.39, ≈2.29 and ≈0.08 respectively. Hence, we may conclude that the hydrogen bond in case of TBAH+2-butanol is stronger as compared to other butanol isomers.

Our interpretation of hydrogen bonding between of IL and butanol isomers (based *V^E^* data) is quite corroborated with our theoretical calculation of hydrogen bonding of IL+butanol isomers. It is noteworthy that the hydroxyl groups of alcohols are interacting with the nitrogen group of ILs (TPAH and TBAH) (Figures S1, S2, S3, S4, S5, S6). According to literature, the negative *V^E^* values are a result of contributions from both the accommodation of organic molecules in the interstice of the IL networks and the ion–dipole interactions between the organic molecules and cation of the ionic liquid [38], [57]. Our experimental results reveal that the negative *V^E^* values for entire composition and theoretical calculation suggested that hydroxyl groups of alcohols are interacting with the cation of ILs, as illustrated in Figures S1, S2, S3, S4, S5, S6. Hence, our results are very well correlated with literature results. Therefore, the absolute value of *V^E^* is an indicative to the difference in the packing efficiency and the interaction intensity. As can be seen from Figure 7, the *V^E^* values for the studied systems follow the sequence: 2-methyl-2-propanol>1-butanol>2-butanol. If only ion–dipole interactions are taken into consideration, the order 1-butanol>2-butanol>2-methyl-2-propanol is understandable. The decreased dielectric constant from 1-butanol (17.8), 2-butanol (16.6) and 2-methyl-2-propanol (10.9) leads to the weaker ion–dipole interaction and in turn resulting in the smaller *V^E^* values. Whereas, if we consider the energies of the hydrogen bond (Table 2), the order for ILs+butanol isomers follows: 2-butanol>1-butanol>2-methyl-2-propanol. Our experimental results suggest the order 2-methyl-2-propanol>1-butanol>2-butanol, which reveal that the interactions are not only due to individual contribution of ion-dipole interaction or H-bonding, but it is the combined effect of both the factors. Whereas, another plausible reason is that the butanol isomers makes it easy to accommodate in the interstice of the IL network, and the higher packing efficiency also leads to the larger *V^E^* values. While, with increase in temperature there is decrease in *V^E^* values in all the systems because at higher temperature the packing efficiency decreases of ILs. On the other hand, the ion-dipole interactions also decrease with the increase in temperature that leads to decrease in *V^E^* values.

Further, for better understanding of the interactions between the tetraalkylammonium hydroxide ILs, we have calculated the Δ*κ_s_*. As seen in Figure 8, Δ*κ_s_* values of tetraalkylammonium hydroxide ILs+butanol isomers are negative over the full composition range at 293.15 K as a function of ILs concentration.The behavior of Δ*κ_s_*, implies that these mixtures are less compressible than the ideal mixture. This is due to closer approach of unlike molecules and a stronger interaction between components of mixtures that leads to a decrease in the compressibility. From Figure 8a, it can be seen that the minimum Δ*κ_s_* values are observed at mole fraction of IL ≈0.4141 for the TPAH+1-butanol system. The negative Δ*κ_s_* values of TPAH+1-butanol are attributed to the strong attractive interactions due to the solvation of the ions in these solvents, over the complete composition range and at all studied temperatures. Similarly, the curves in Figure 8 (c and d), show that the Δ*κ_s_* values for the 2-butanol or 2-methyl-2-propanol+TPAH systems are negative over the complete composition range and at all studied temperatures, except at the higher temperatures (308.15 and 313.15 K) for ≈0.8100 to 0.9999 composition range. The minimum is approached at mole fraction of IL ≈0.4171, ≈0.3272 and ≈0.3138 for the TPAH+1-butanol, TPAH+2-butanol and TPAH+2-methyl-2-propanol systems at all temperatures, respectively. The negative Δ*κ_s_* values attributed to the strong attractive interactions between the molecules of the components. The negative values of Δ*κ_s_* of the TPAH with butanol isomers imply that solvent molecules around solute are less compressible than the solvent molecules in the bulk solutions. Whereas on further addition of IL, there is decrease in the compressibility graph at all studied temperature ranges. This might be due to the decreased attraction between IL and butanol isomers in IL rich concentration region. Additionally, for the 2-butanol and 2-methyl-2-propanol, there are positive Δκ_s_ values at higher temperatures, this is might be again due to decrease in attraction of TPAH and alcohol molecules in the IL-rich concentration region, since the interaction between the ILs increases and whereas decreases in case of IL and alcohols.

Figure 9 depicts the negative Δ*κ_s_* values of all TBAH+butanol isomers over the full composition range at 293.15 K. The curves in Figure 9 show that the Δκ_s_ values for the 1-butanol or 2-butanol or 2-methyl-2-propanol systems are negative over the complete composition range at low temperature. The minimum is approached at mole fractions of IL ≈0.4787, ≈0.4604 and ≈0.1985 for the TBAH+1-butanol, TBAH+2-butanol and TBAH+2-methyl-2-propanol systems, respectively. Our results show that for all the system, TBAH+butanol isomers shows the positive Δ*κ_s_* values in the IL-rich region at the higher temperatures. These results are very similar with the TPAH+alcohols at higher temperature, which might be due to the decrease in the attraction of TBAH and alcohol molecules in the IL-rich concentration region due to the increased interaction between the ILs and the decreased interaction between IL and alcohols. Obviously, the Δ*κ_s_* values in the ILs+2-methyl-2-propanol mixture shows more negative values of Δ*κ_s_* than the ILs+2-butanol and ILs+1-butanol mixtures at 293.15 K over the entire concentration range (Figure 10), implying that in the 2-methyl-2-propanol there is strong ion-dipole interactions and packing effects with ILs as compared to 2-butanol and 1-butanol. The magnitude and sign of Δ*κ_s_* values are a reflection of the type of interactions taking place in the mixture, which reveals that Δ*κ_s_* values are more negative for TBAH+2-methyl-2-propanol (Δ*κ_s_* = −211.351TPa^−1^ at *x*_1_ = 0.1985), than TBAH+1-butanol (Δ*κ_s_* = −168.519TPa^−1^ at *x*_1_ = 0.4787), and least is for TBAH+2-butanol (Δ*κ_s_* = −144.998TPa^−1^ at *x*_1_ = 0.4604). For TPAH+2-methyl-2-propanol (Δ*κ_s_* = −264.189TPa^−1^ at *x*_1_ = 0.3115), TPAH+1-butanol (Δ*κ_s_* = −222.322TPa^−1^ at *x*_1_ = 0.3218), and TPAH+2-butanol (Δ*κ_s_* = −153.949TPa^−1^ at *x*_1_ = 0.0.3244). TPAH+butanol isomers have more negative Δ*κ_s_* values then TBAH+butanol isomers this is might be due to the steric hindrance created by the long chain cation of TBAH, which reduces the interaction magnitude between TBAH and alcohols.

However, after close look about the physical properties of alcohols with ILs, we observed that hydroxyl position of alcohols are playing important role in addition to the cation chain length of the ILs. ILs (TPAH or TBAH) interact strongly with the 2-methyl-2-propanol as compared to the 2-butanol and 1-butanol, this is might be due to more+I-effect of 2-methyl-2-propanol, which increases its tendency to interact with ILs more strongly as compared to 2-butanol and 1-butanol. Also, 1-butanol interacts more strongly as compared to 2-butanol with ILs, which might be due to the steric hindrance the interaction of 2-butanol decreases as compared to 1-butanol. However, there is decrease in *V* ^E^ and Δ*κ_s_* values in all the system due to increase in temperature; this might be due to strong self-association between the alcohol molecules that prevents the alcohol-IL strong interactions. Our experimental results of *V* ^E^ values are very well supported with literature [38], [46], [47]. Wen-Lu Weng [47], showed the interactions of anisole with 2-butanol and 2-methyl-2-propanol, author observed that *V* ^E^ values of the 2-methyl-2-propanol is more negative than 2-butanol. Additionally, the *V* ^E^ values increases (less negative) with increase in temperature. Further, Qian et al. [38], showed that 1-methylimidazolium acetate IL interacts with methanol, ethanol, 1-propanol and 1-butanol, it was observed that in all the systems *V* ^E^ values increase with increase in the temperature. Moreover, during the interaction of formamide with 1-butanol negative *V* ^E^ values have been observed, whereas interaction of formamide with 2-butanol results in positive *V* ^E^ values [46]. These all results by various authors support our above results explanation that interactions between ILs+alcohols depend upon the position of hydroxyl group. Therefore, the physicochemical properties of ILs are quite sensitive toward the structure and nature of interacting molecules.

### Conclusion

We have performed and compared thermophysical properties of binary mixtures of tetraalkylammonium hydroxide based ILs with butanol isomers over the whole composition range at various temperatures (293.15 to 313.15 K, in steps of 5 K). To obtain a more detailed picture of the molecular interactions, we measured temperature dependence properties of *ρ* and *u* for ILs with butanol isomers over the whole composition range at various temperatures. The *ρ* and *u* values increase with the increasing the cation alkyl chain length of ILs. Our results reveal that the position of hydroxyl group in alcohols leads to alteration of the thermophysical properties of ILs. To measure the non-ideality of the mixtures, we determined *V^E^* and Δ*κ_s_* at each temperature as a function of IL concentration. The predicted properties were correlated by the Redlich-Kister type equation. Our studies demonstrate that there is decrease in *V* ^E^ and Δ*κ_s_* values in all the systems due to increase in temperature; this might be due to strong self-association between the alcohol molecules that prevents the alcohol-IL strong interactions. Additionally, according to the theoretical calculations obtained by HyperChem 7, the energy of hydrogen bond is more for low alkyl chain ILs (TPAH) as compared to higher alkyl chain ILs (TBAH) with alcohols. Molecular interactions such as ion-dipole and hydrogen bonding between the butanol isomers and alkyl chain of ILs are suggested to be mainly responsible for variation in the thermophysical parameters. Our findings provide better molecular interactions for the mixing of the solvents and better analysis of the solvation process.

## Supporting Information

Figure S1 **Schematic depiction of the hydrogen bonding interaction between TPAH and 1-butanol molecules, which is predicted by a semiempirical calculation with the help of HyperChem 7.**
(TIF)

Figure S2 **Schematic depiction of the hydrogen bonding interaction between TPAH and 2-butanol molecules, which is predicted by a semiempirical calculation with the help of HyperChem 7.**
(TIF)

Figure S3 **Schematic depiction of the hydrogen bonding interaction between TPAH and 2-methyl-2-propanol molecules, which is predicted by a semiempirical calculation with the help of HyperChem 7.**
(TIF)

Figure S4 **Schematic depiction of the hydrogen bonding interaction between TBAH and 1-butanol molecules, which is predicted by a semiempirical calculation with the help of HyperChem 7.**
(TIF)

Figure S5 **Schematic depiction of the hydrogen bonding interaction between TBAH and 2-butanol molecules, which is predicted by a semiempirical calculation with the help of HyperChem 7.**
(TIF)

Figure S6 **Schematic depiction of the hydrogen bonding interaction between TBAH and 2-methyl-2-propanolmolecules, which is predicted by a semiempirical calculation with the help of HyperChem 7.**
(TIF)

File S1 **Supporting Tables.** Table S1. Mole fraction (*x*_1_) of IL, density (ρ), ultrasonic sound velocity (*u*), Excess molar volumes (*V^E^*), isentropic compressibility (*κ_s_*), and deviation in isentropic compressibility (Δ*κ_s_*) for the systems of tetraalkylammonium hydroxide IL with butanol isomers at T = 293.15, 298.15, 303.15, 308.15 and 313.15 K and at atmospheric pressure. Table S2. Estimated Parameters of eq 1 and Standard Deviation σ, for the Systems of ILs with butanol isomers as Function of Temperature.
(DOCX)
