# Supplementary figures and images for: Influence of Hydroxyl Group Position and Temperature on Thermophysical Properties of Tetraalkylammonium Hydroxide Ionic Liquids with Alcohols
Source: PLoS One. 2014 Jan 29;9(1):e86530. doi: 10.1371/journal.pone.0086530 (PMC3906063; doi:10.1371/journal.pone.0086530)

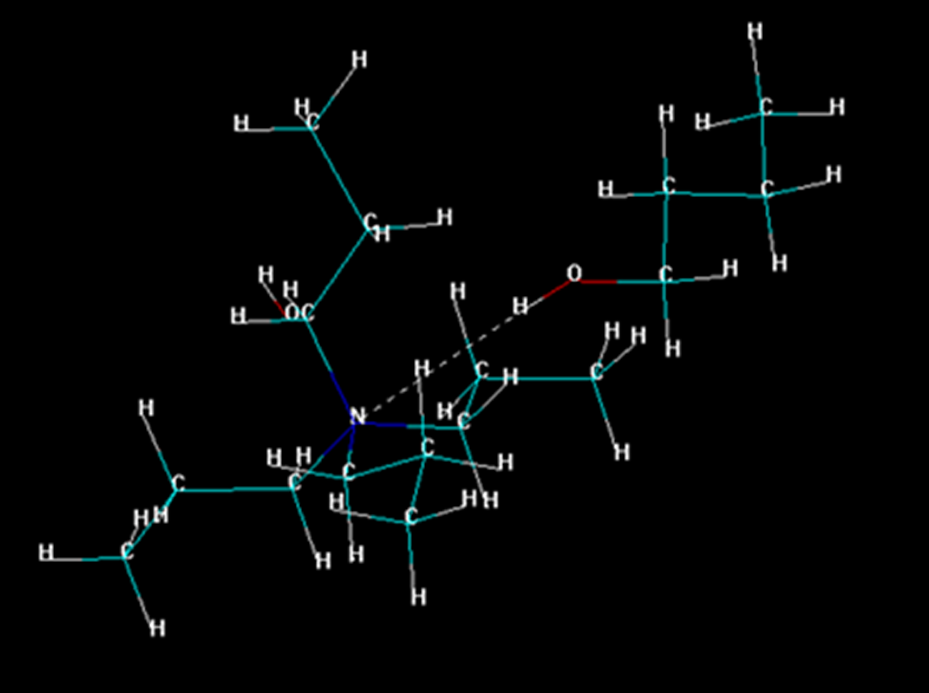

Supplement: Figure S1 — Schematic depiction of the hydrogen bonding interaction between TPAH and 1-butanol molecules, which is predicted by a semiempirical calculation with the help of HyperChem 7. (TIF) [file pone.0086530.s001.tif]

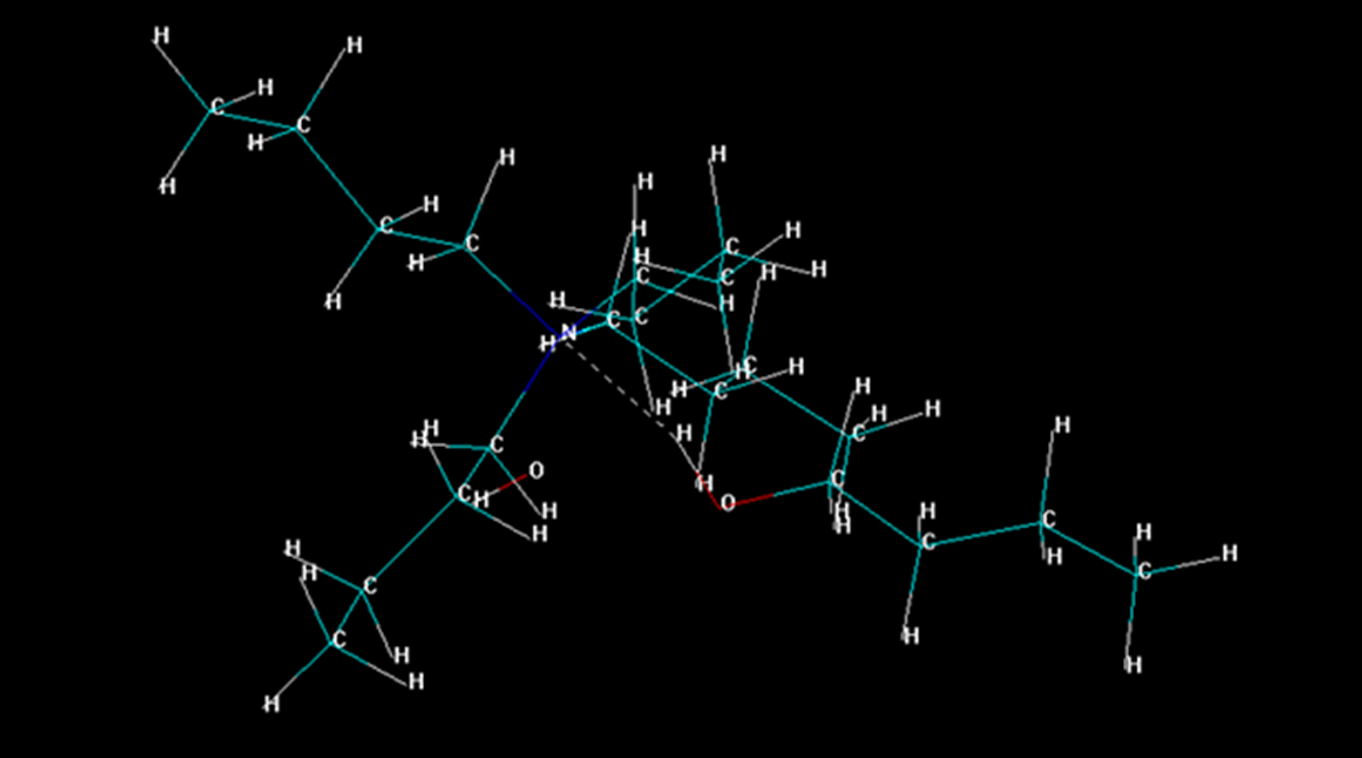

Supplement: Figure S2 — Schematic depiction of the hydrogen bonding interaction between TPAH and 2-butanol molecules, which is predicted by a semiempirical calculation with the help of HyperChem 7. (TIF) [file pone.0086530.s002.tif]

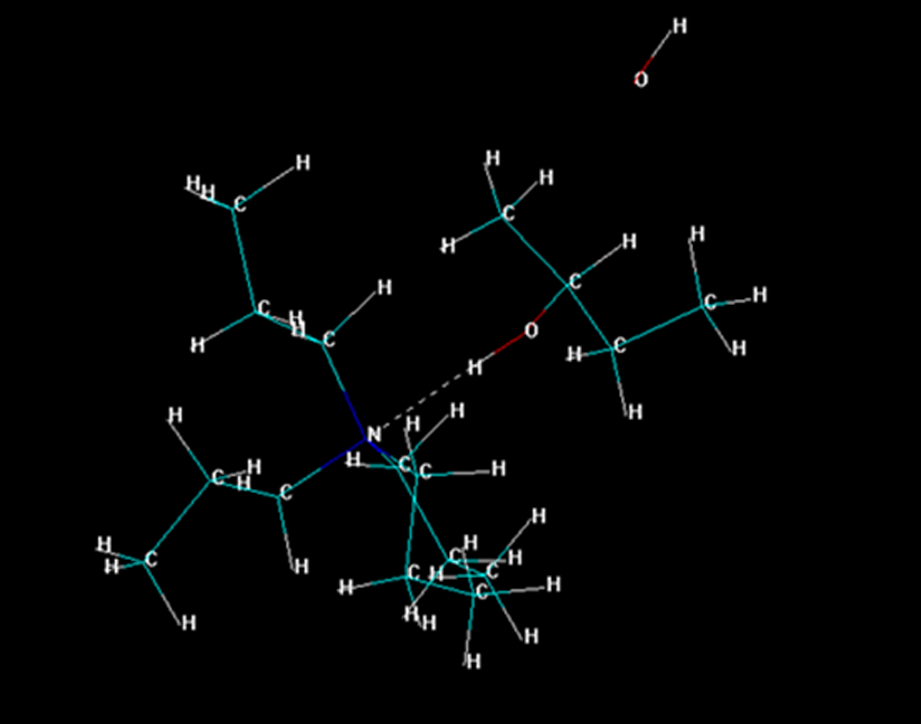

Supplement: Figure S3 — Schematic depiction of the hydrogen bonding interaction between TPAH and 2-methyl-2-propanol molecules, which is predicted by a semiempirical calculation with the help of HyperChem 7. (TIF) [file pone.0086530.s003.tif]

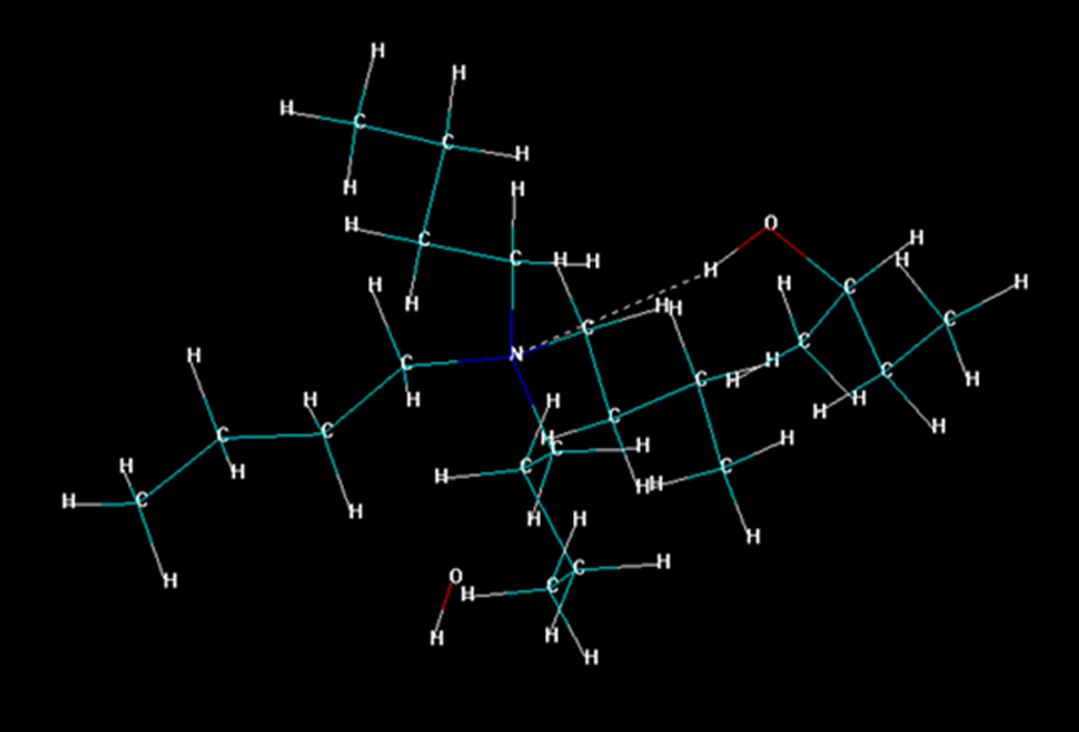

Supplement: Figure S4 — Schematic depiction of the hydrogen bonding interaction between TBAH and 1-butanol molecules, which is predicted by a semiempirical calculation with the help of HyperChem 7. (TIF) [file pone.0086530.s004.tif]

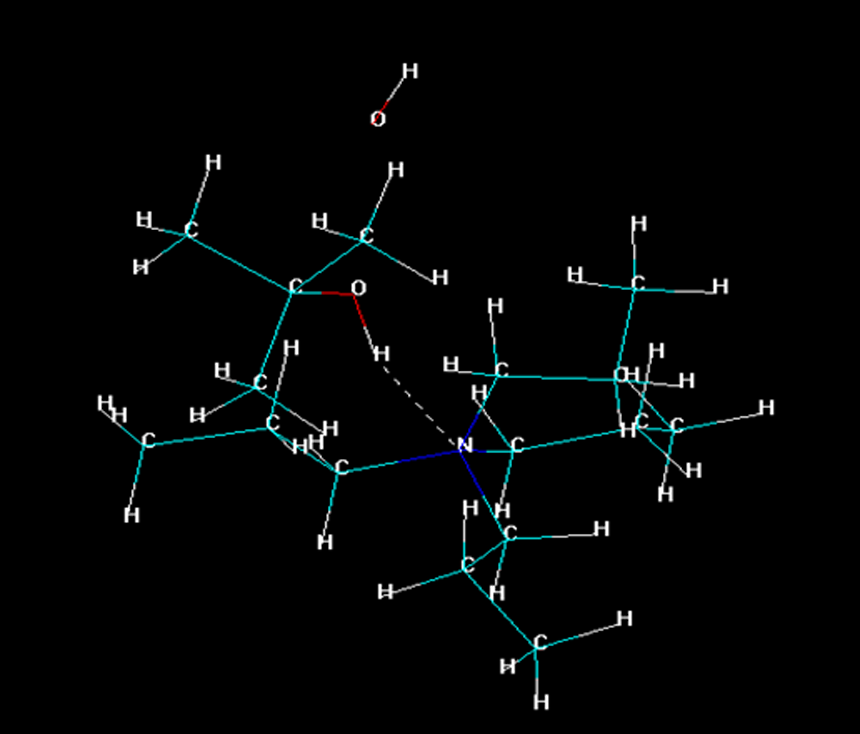

Supplement: Figure S5 — Schematic depiction of the hydrogen bonding interaction between TBAH and 2-butanol molecules, which is predicted by a semiempirical calculation with the help of HyperChem 7. (TIF) [file pone.0086530.s005.tif]

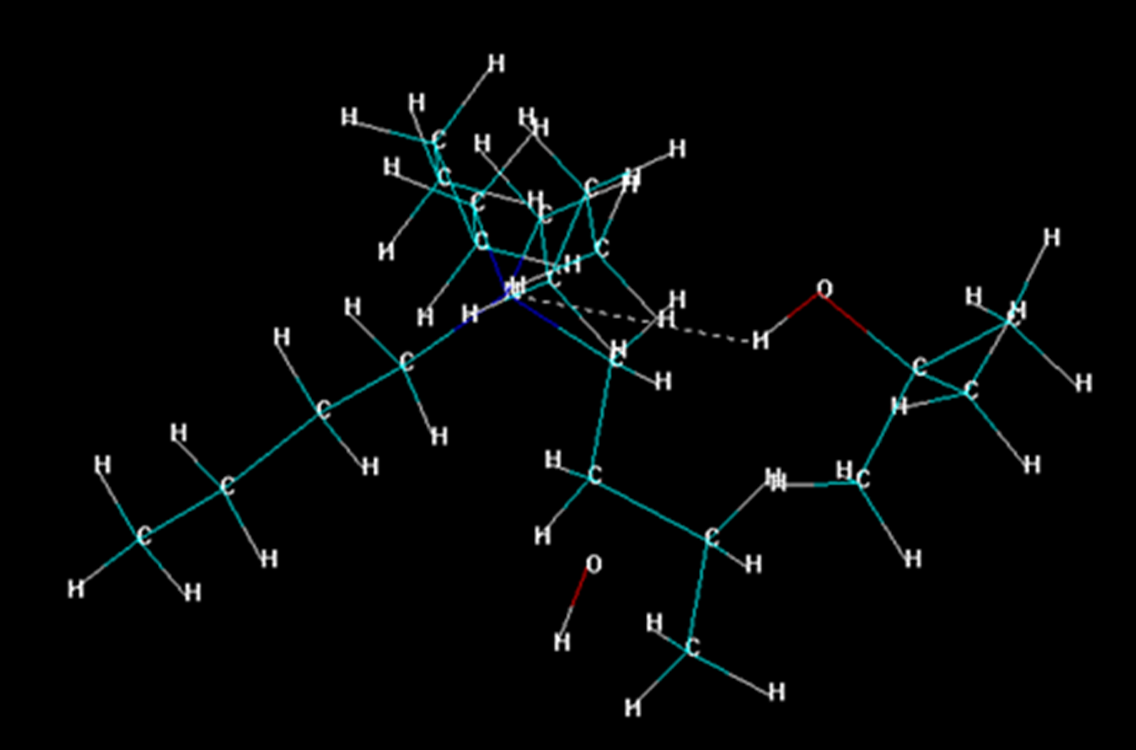

Supplement: Figure S6 — Schematic depiction of the hydrogen bonding interaction between TBAH and 2-methyl-2-propanolmolecules, which is predicted by a semiempirical calculation with the help of HyperChem 7. (TIF) [file pone.0086530.s006.tif]
