# Supplementary material for: Influence of Hydroxyl Group Position and Temperature on Thermophysical Properties of Tetraalkylammonium Hydroxide Ionic Liquids with Alcohols
Source: PLoS One. 2014 Jan 29;9(1):e86530. doi: 10.1371/journal.pone.0086530 (PMC3906063; doi:10.1371/journal.pone.0086530)
Supplement: File S1 — Supporting Tables. Table S1. Mole fraction (x 1) of IL, density (ρ), ultrasonic sound velocity (u), Excess molar volumes (VE), isentropic compressibility (κs), and deviation in isentropic compressibility (Δκs) for the systems of tetraalkylammonium hydroxide IL with butanol isomers at T = 293.15, 298.15, 303.15, 308.15 and 313.15 K and at atmospheric pressure. Table S2. Estimated Parameters of eq 1 and Standard Deviation σ, for the Systems of ILs with butanol isomers as Function of Temperature. (DOCX) [file pone.0086530.s007.docx]

**Supporting Information**

**Influence of Hydroxyl Group Position and Temperature on Thermophysical Properties of Tetraalkylammonium Hydroxide Ionic Liquids with Alcohols**

**Pankaj Attri^1^*, K Y Baik^1^, Pannuru Venkatesu^2^, In Tae Kim^3^, Eun Ha Choi*^1^**

^1^Plasma Bioscience Research Center / Department of Electrical and Biological Physics, Kwangwoon University, Seoul, Korea, 139-791

^2^Department of Chemistry, University of Delhi, Delhi – 110 007, India

^3^Department of Chemistry, Kwangwoon University, Seoul, Korea 139-701.

**Table S1 Page 2-11**

**Table S2 Page 12-13**

**Table S1.** Mole fraction (*x*_1_) of IL, density (ρ), ultrasonic sound velocity (*u*), Excess molar volumes (*V^E^*), isentropic compressibility (*κ_s_*), and deviation in isentropic compressibility (Δ *κ_s_*) for the systems of tetraalkylammonium hydroxide IL with butanol isomers at T = 293.15, 298.15, 303.15, 308.15 and 313.15 K and at atmospheric pressure.

| x_1_ | ρ/g.cm^-3^ | u/m.s^-1^ | *V^E^* /cm^3^.mol^-1^ | | *κ_s_*/T.Pa^- 1^ | | ∆*κ_s_*/T.Pa^-1^ | | | |
| --- | --- | --- | --- | --- | --- | --- | --- | --- | --- | --- |
| TPAH with 1-butanol at 293.15 K | | | | | | | | | |  |
| 0.0000 | 0.80980 | 1257 | 0 | | 782 | | 0 | | |  |
| 0.0608 | 0.85034 | 1326 | -1.955 | | 668 | | -84.2 | | |  |
| 0.1056 | 0.87097 | 1375 | -2.624 | | 607 | | -124.7 | | |  |
| 0.1749 | 0.89887 | 1462 | -3.571 | | 520 | | -177.7 | | |  |
| 0.2378 | 0.91943 | 1534 | -4.204 | | 462 | | -206.2 | | |  |
| 0.3200 | 0.94294 | 1614 | -5.014 | | 407 | | -222.0 | | |  |
| 0.4145 | 0.96314 | 1701 | -5.520 | | 359 | | -225.3 | | |  |
| 0.5621 | 0.98486 | 1787 | -5.655 | | 318 | | -196.1 | | |  |
| 0.7212 | 0.99823 | 1846 | -4.927 | | 294 | | -144.2 | | |  |
| 0.8139 | 1.00220 | 1861 | -4.050 | | 288 | | -105.8 | | |  |
| 0.9187 | 1.00424 | 1851 | -2.698 | | 291 | | -53.4 | | |  |
| 0.9835 | 1.00458 | 1813 | -1.707 | | 303 | | -10.4 | | |  |
| 1.0000 | 0.99748 | 1810 | 0 | | 305 | | 0 | | |  |
| TPAH with 1-butanol at 298.15 K | | | | | | | | | |  |
| 0.0000 | 0.80567 | 1240 | 0 | | 807 | | 0 | | |  |
| 0.0608 | 0.84343 | 1300 | -1.653 | | 701 | | -75.6 | | |  |
| 0.1056 | 0.86457 | 1341 | -2.382 | | 642 | | -112.5 | | |  |
| 0.1749 | 0.89109 | 1404 | -3.160 | | 569 | | -151.2 | | |  |
| 0.2378 | 0.91155 | 1461 | -3.767 | | 513 | | -175.7 | | |  |
| 0.3200 | 0.93536 | 1534 | -4.597 | | 454 | | -194.1 | | |  |
| 0.4145 | 0.95517 | 1610 | -5.020 | | 404 | | -197.4 | | |  |
| 0.5621 | 0.97706 | 1700 | -5.122 | | 354 | | -174.2 | | |  |
| 0.7212 | 0.99142 | 1765 | -4.483 | | 324 | | -125.2 | | |  |
| 0.8139 | 0.99630 | 1794 | -3.730 | | 312 | | -91.1 | | |  |
| 0.9187 | 0.99859 | 1810 | -2.387 | | 306 | | -45.0 | | |  |
| 0.9835 | 0.99904 | 1817 | -1.392 | | 303 | | -15.3 | | |  |
| 1.0000 | 0.99358 | 1801 | 0 | | 310 | | 0 | | |  |
| TPAH with 1-butanol at 303.15 K | | | | | | | | | |  |
| 0.0000 | 0.80195 | 1222 | 0 | | 835 | | 0 | | |  |
| 0.0608 | 0.83733 | 1270 | -1.346 | | 740 | | -63.3 | | |  |
| 0.1056 | 0.85795 | 1308 | -1.980 | | 681 | | -99.4 | | |  |
| 0.1749 | 0.88570 | 1371 | -2.850 | | 600 | | -143.7 | | |  |
| 0.2378 | 0.90700 | 1422 | -3.514 | | 545 | | -165.6 | | |  |
| 0.3200 | 0.93043 | 1490 | -4.232 | | 484 | | -184.5 | | |  |
| 0.4145 | 0.95110 | 1565 | -4.697 | | 429 | | -189.8 | | |  |
| 0.5621 | 0.97410 | 1653 | -4.850 | | 375 | | -166.9 | | |  |
| 0.7212 | 0.98860 | 1719 | -4.118 | | 342 | | -117.4 | | |  |
| 0.8139 | 0.99401 | 1756 | -3.391 | | 326 | | -84.8 | | |  |
| 0.9187 | 0.99646 | 1778 | -2.005 | | 317 | | -39.2 | | |  |
| 0.9835 | 0.99725 | 1791 | -1.034 | | 312 | | -10.4 | | |  |
| 1.0000 | 0.99360 | 1790 | 0 | | 314 | | 0 | | |  |
| TPAH with 1-butanol at 308.15 K | | | | | | | | | |  |
| 0.0000 | 0.79821 | 1207 | 0 | | 860 | | 0 | | |  |
| 0.0608 | 0.83202 | 1245 | -1.159 | | 774 | | -52.7 | | |  |
| 0.1056 | 0.85257 | 1279 | -1.775 | | 716 | | -86.5 | | |  |
| 0.1749 | 0.88087 | 1339 | -2.698 | | 633 | | -132.8 | | |  |
| 0.2378 | 0.90175 | 1383 | -3.294 | | 579 | | -151.9 | | |  |
| 0.3200 | 0.92543 | 1448 | -4.021 | | 515 | | -172.1 | | |  |
| 0.4145 | 0.94621 | 1523 | -4.470 | | 455 | | -180.7 | | |  |
| 0.5621 | 0.96968 | 1614 | -4.643 | | 396 | | -160.4 | | |  |
| 0.7212 | 0.98459 | 1676 | -3.921 | | 361 | | -108.7 | | |  |
| 0.8139 | 0.98991 | 1709 | -3.144 | | 346 | | -74.4 | | |  |
| 0.9187 | 0.99277 | 1736 | -1.794 | | 334 | | -29.1 | | |  |
| 0.9835 | 0.99359 | 1765 | -0.804 | | 323 | | -5.4 | | |  |
| 1.0000 | 0.99111 | 1777 | 0 | | 320 | | 0 | | |  |
| TPAH with 1-butanol at 313.15 K | | | | | | | | | |  |
| 0.0000 | 0.79420 | 1190 | 0 | | 889 | | 0 | | |  |
| 0.0608 | 0.82408 | 1224 | -0.718 | | 809 | | -45.8 | | |  |
| 0.1056 | 0.84471 | 1254 | -1.355 | | 752 | | -77.6 | | |  |
| 0.1749 | 0.87316 | 1306 | -2.311 | | 671 | | -119.3 | | |  |
| 0.2378 | 0.89421 | 1349 | -2.936 | | 614 | | -141.1 | | |  |
| 0.3200 | 0.91811 | 1416 | -3.702 | | 543 | | -165.6 | | |  |
| 0.4145 | 0.93918 | 1487 | -4.196 | | 481 | | -173.9 | | |  |
| 0.5621 | 0.96308 | 1573 | -4.437 | | 419 | | -152.2 | | |  |
| 0.7212 | 0.97768 | 1634 | -3.653 | | 383 | | -98.7 | | |  |
| 0.8139 | 0.98306 | 1672 | -2.875 | | 364 | | -65.5 | | |  |
| 0.9187 | 0.98615 | 1710 | -1.550 | | 347 | | -23.4 | | |  |
| 0.9835 | 0.98707 | 1755 | -0.569 | | 329 | | -4.8 | | |  |
| 1.0000 | 0.98576 | 1769 | 0 | | 324 | | 0 | | |  |
| TPAH with 2-butanol at 293.15 K | | | | | | | | | |  |
| 0.0000 | 0.80709 | 1229 | 0 | | 820 | | 0 | | |  |
| 0.0612 | 0.84087 | 1274 | -1.141 | | 732 | | -57.2 | | |  |
| 0.1110 | 0.86109 | 1313 | -1.537 | | 673 | | -90.9 | | |  |
| 0.1795 | 0.88594 | 1363 | -2.102 | | 607 | | -122.2 | | |  |
| 0.2390 | 0.90379 | 1407 | -2.429 | | 559 | | -140.1 | | |  |
| 0.3250 | 0.92627 | 1466 | -2.884 | | 502 | | -153.3 | | |  |
| 0.4157 | 0.94539 | 1520 | -3.182 | | 457 | | -151.9 | | |  |
| 0.6540 | 0.97856 | 1623 | -2.990 | | 388 | | -100.6 | | |  |
| 0.7220 | 0.98492 | 1640 | -2.733 | | 377 | | -76.8 | | |  |
| 0.8150 | 0.99196 | 1676 | -2.241 | | 358 | | -48.2 | | |  |
| 0.9190 | 0.99717 | 1731 | -1.359 | | 335 | | -19.4 | | |  |
| 0.9890 | 1.00019 | 1786 | -0.740 | | 312 | | -5.1 | | |  |
| 1.0000 | 0.99748 | 1812 | 0 | | 313 | | 0 | | |  |
| TPAH with 2-butanol at 298.15 K | | | | | | | | | |  |
| 0.0000 | 0.80267 | 1211 | 0 | | 850 | | 0 | | |  |
| 0.0612 | 0.83529 | 1250 | -0.983 | | 766 | | -50.6 | | |  |
| 0.1110 | 0.85584 | 1286 | -1.392 | | 706 | | -83.3 | | |  |
| 0.1795 | 0.88054 | 1332 | -1.898 | | 640 | | -113.0 | | |  |
| 0.2390 | 0.89834 | 1377 | -2.184 | | 587 | | -133.4 | | |  |
| 0.3250 | 0.92130 | 1433 | -2.651 | | 528 | | -145.7 | | |  |
| 0.4157 | 0.94051 | 1489 | -2.907 | | 479 | | -145.8 | | |  |
| 0.6540 | 0.97467 | 1587 | -2.725 | | 407 | | -89.0 | | |  |
| 0.7220 | 0.98149 | 1613 | -2.508 | | 391 | | -68.2 | | |  |
| 0.8150 | 0.98858 | 1651 | -1.974 | | 371 | | -38.5 | | |  |
| 0.9190 | 0.99467 | 1720 | -1.203 | | 340 | | -13.7 | | |  |
| 0.9890 | 0.99786 | 1789 | -0.586 | | 313 | | -2.5 | | |  |
| 1.0000 | 0.99594 | 1801 | 0 | | 310 | | 0 | | |  |
| TPAH with 2-butanol at 303.15 K | | | | | | | | | |  |
| 0.0000 | 0.79876 | 1193 | 0 | | 880 | | 0 | | |  |
| 0.0612 | 0.83047 | 1230 | -0.866 | | 796 | | -49.2 | | |  |
| 0.1110 | 0.85122 | 1260 | -1.285 | | 740 | | -76.9 | | |  |
| 0.1795 | 0.87594 | 1303 | -1.776 | | 672 | | -106.0 | | |  |
| 0.2390 | 0.89391 | 1344 | -2.063 | | 619 | | -125.7 | | |  |
| 0.3250 | 0.91654 | 1399 | -2.458 | | 557 | | -138.6 | | |  |
| 0.4157 | 0.93599 | 1455 | -2.715 | | 504 | | -140.2 | | |  |
| 0.6540 | 0.97082 | 1550 | -2.550 | | 429 | | -81.2 | | |  |
| 0.7220 | 0.97764 | 1581 | -2.308 | | 409 | | -62.4 | | |  |
| 0.8150 | 0.98507 | 1617 | -1.803 | | 388 | | -30.7 | | |  |
| 0.9190 | 0.99149 | 1690 | -1.060 | | 353 | | -7.2 | | |  |
| 0.9890 | 0.99509 | 1752 | -0.504 | | 327 | | 6.9 | | |  |
| 1.0000 | 0.99360 | 1790 | 0 | | 314 | | 0 | | |  |
| TPAH with 2-butanol at 308.15 K | | | | | | | | | |  |
| 0.0000 | 0.79446 | 1175 | 0 | | 912 | | 0 | | |  |
| 0.0612 | 0.82503 | 1206 | -0.718 | | 833 | | -42.2 | | |  |
| 0.1110 | 0.84624 | 1230 | -1.178 | | 781 | | -65.3 | | |  |
| 0.1795 | 0.87077 | 1272 | -1.620 | | 709 | | -96.2 | | |  |
| 0.2390 | 0.88913 | 1312 | -1.938 | | 653 | | -116.6 | | |  |
| 0.3250 | 0.91179 | 1368 | -2.305 | | 586 | | -133.1 | | |  |
| 0.4157 | 0.93138 | 1422 | -2.546 | | 531 | | -134.3 | | |  |
| 0.6540 | 0.96700 | 1514 | -2.415 | | 451 | | -73.1 | | |  |
| 0.7220 | 0.97393 | 1545 | -2.168 | | 430 | | -53.9 | | |  |
| 0.8150 | 0.98157 | 1584 | -1.666 | | 406 | | -22.7 | | |  |
| 0.9190 | 0.98807 | 1655 | -0.901 | | 369 | | 2.4 | | |  |
| 0.9890 | 0.99162 | 1723 | -0.308 | | 339 | | 14.1 | | |  |
| 1.0000 | 0.99111 | 1779 | 0 | | 319 | | 0 | | |  |
| TPAH with 2-butanol at 313.15 K | | | | | | | | | |  |
| 0.0000 | 0.79007 | 1157 | 0 | 946 | | 0 | | |  |  |
| 0.0612 | 0.81905 | 1183 | -0.547 | 872 | | -35.3 | | |  |  |
| 0.1110 | 0.8405 | 1204 | -1.049 | 819 | | -57.1 | | |  |  |
| 0.1795 | 0.86495 | 1245 | -1.494 | 746 | | -88.0 | | |  |  |
| 0.2390 | 0.88358 | 1282 | -1.856 | 689 | | -108.4 | | |  |  |
| 0.3250 | 0.90607 | 1338 | -2.213 | 617 | | -127.0 | | |  |  |
| 0.4157 | 0.9256 | 1390 | -2.455 | 559 | | -128.1 | | |  |  |
| 0.6540 | 0.96103 | 1484 | -2.304 | 472 | | -67.0 | | |  |  |
| 0.7220 | 0.96795 | 1513 | -2.053 | 451 | | -45.6 | | |  |  |
| 0.8150 | 0.97561 | 1554 | -1.551 | 424 | | -15.2 | | |  |  |
| 0.9190 | 0.98214 | 1628 | -0.787 | 384 | | 9.3 | | |  |  |
| 0.9890 | 0.98565 | 1699 | -0.182 | 351 | | 20.2 | | |  |  |
| 1.0000 | 0.98576 | 1769 | 0 | 324 | | 0 | | |  |  |
| TPAH with 2-methyl-2-propanol at 293.15 K | | | | | | | | | |  |
| 0.0000 | 0.78576 | 1145 | 0 | 971 | | 0 | | |  |  |
| 0.0568 | 0.83669 | 1201 | -3.191 | 828 | | -105.0 | | |  |  |
| 0.1056 | 0.86224 | 1254 | -4.108 | 737 | | -163.6 | | |  |  |
| 0.1709 | 0.89179 | 1314 | -5.172 | 649 | | -208.3 | | |  |  |
| 0.2440 | 0.91927 | 1391 | -6.127 | 562 | | -246.2 | | |  |  |
| 0.3120 | 0.94058 | 1460 | -6.836 | 499 | | -264.5 | | |  |  |
| 0.4145 | 0.96606 | 1546 | -7.560 | 433 | | -261.8 | | |  |  |
| 0.6600 | 0.99954 | 1659 | -7.136 | 364 | | -168.0 | | |  |  |
| 0.7132 | 1.00222 | 1664 | -6.531 | 360 | | -135.9 | | |  |  |
| 0.8069 | 1.00545 | 1692 | -5.282 | 347 | | -86.7 | | |  |  |
| 0.9117 | 1.00586 | 1737 | -3.368 | 329 | | -34.7 | | |  |  |
| 0.9775 | 1.00559 | 1799 | -2.068 | 307 | | -13.2 | | |  |  |
| 1.0000 | 0.99748 | 1812 | 0 | 305 | | 0 | | |  |  |
| TPAH with 2-methyl-2-propanol at 298.15 K | | | | | | | |  |  |  |
| 0.0000 | 0.78080 | 1123 | 0 | 102 | | 0 | | |  |  |
| 0.0568 | 0.82931 | 1156 | -0.238 | 901 | | -74.5 | | |  |  |
| 0.1056 | 0.85384 | 1197 | -0.581 | 817 | | -123.8 | | |  |  |
| 0.1709 | 0.88065 | 1259 | -0.878 | 716 | | -179.2 | | |  |  |
| 0.2440 | 0.9075 | 1339 | -1.191 | 614 | | -229.3 | | |  |  |
| 0.3120 | 0.92856 | 1408 | -1.442 | 543 | | -252.3 | | |  |  |
| 0.4145 | 0.95510 | 1483 | -1.665 | 475 | | -247.5 | | |  |  |
| 0.6600 | 0.98890 | 1602 | -2.131 | 394 | | -155.6 | | |  |  |
| 0.7132 | 0.99330 | 1612 | -2.409 | 387 | | -124.6 | | |  |  |
| 0.8069 | 0.99732 | 1643 | -2.47 | 372 | | -74.0 | | |  |  |
| 0.9117 | 1.00162 | 1688 | -2.473 | 350 | | -21.5 | | |  |  |
| 0.9775 | 1.00192 | 1761 | -2.362 | 322 | | -3.7 | | |  |  |
| 1.0000 | 0.99594 | 1801 | -2.167 | 310 | | 0 | | |  |  |
| TPAH with 2-methyl-2-propanol at 303.15 K | | | | | | | |  |  |  |
| 0.0000 | 0.77531 | 1102 | 0 | 106 | | 0 | | |  |  |
| 0.0568 | 0.81532 | 1131 | -1.884 | 958 | | -61.8 | | |  |  |
| 0.1056 | 0.84137 | 1162 | -2.805 | 880 | | -103.3 | | |  |  |
| 0.1709 | 0.87081 | 1223 | -3.763 | 767 | | -167.1 | | |  |  |
| 0.2440 | 0.89859 | 1294 | -4.638 | 664 | | -215.2 | | |  |  |
| 0.3120 | 0.92044 | 1351 | -5.298 | 595 | | -234.0 | | |  |  |
| 0.4145 | 0.94641 | 1429 | -5.891 | 517 | | -235.2 | | |  |  |
| 0.6600 | 0.98295 | 1539 | -5.402 | 429 | | -139.3 | | |  |  |
| 0.7132 | 0.98727 | 1547 | -4.950 | 423 | | -105.7 | | |  |  |
| 0.8069 | 0.99243 | 1585 | -3.843 | 401 | | -57.7 | | |  |  |
| 0.9117 | 0.99608 | 1651 | -2.333 | 368 | | -11.8 | | |  |  |
| 0.9775 | 0.99663 | 1721 | -1.084 | 339 | | 7.7 | | |  |  |
| 1.0000 | 0.99360 | 1790 | 0 | 314 | | 0 | | |  |  |
| TPAH with 2-methyl-2-propanol at 308.15 K | | | | | | | | | |  |
| 0.0000 | 0.76481 | 1080 | 0 | 112 | | 0 | | |  |  |
| 0.0568 | 0.80324 | 1101 | -1.648 | 103 | | -48.7 | | |  |  |
| 0.1056 | 0.82939 | 1126 | -2.526 | 950 | | -86.1 | | |  |  |
| 0.1709 | 0.86029 | 1177 | -3.588 | 839 | | -145.2 | | |  |  |
| 0.2440 | 0.88783 | 1239 | -4.331 | 733 | | -192.1 | | |  |  |
| 0.3120 | 0.90956 | 1292 | -4.873 | 658 | | -212.6 | | |  |  |
| 0.4145 | 0.93617 | 1372 | -5.398 | 567 | | -221.3 | | |  |  |
| 0.6600 | 0.97540 | 1487 | -4.933 | 463 | | -128.4 | | |  |  |
| 0.7132 | 0.98028 | 1488 | -4.493 | 461 | | -88.3 | | |  |  |
| 0.8069 | 0.98683 | 1531 | -3.491 | 432 | | -41.5 | | |  |  |
| 0.9117 | 0.99137 | 1600 | -2.004 | 394 | | 4.1 | | |  |  |
| 0.9775 | 0.99216 | 1685 | -0.710 | 355 | | 17.8 | | |  |  |
| 1.0000 | 0.99111 | 1779 | 0 | 319 | | 0 | | |  |  |
| TPAH with 2-methyl-2-propanol at 313.15 K | | | | | | | |  |  |  |
| 0.0000 | 0.76507 | 1059 | 0 | 117 | | 0 | | |  |  |
| 0.0568 | 0.79676 | 1083 | -0.856 | 107 | | -48.4 | | |  |  |
| 0.1056 | 0.82126 | 1101 | -1.567 | 100 | | -72.0 | | |  |  |
| 0.1709 | 0.84845 | 1149 | -2.191 | 892 | | -129.7 | | |  |  |
| 0.2440 | 0.87488 | 1208 | -2.816 | 783 | | -177.6 | | |  |  |
| 0.3120 | 0.89558 | 1263 | -3.235 | 699 | | -203.7 | | |  |  |
| 0.4145 | 0.92137 | 1338 | -3.649 | 606 | | -210.8 | | |  |  |
| 0.6600 | 0.96167 | 1446 | -3.325 | 497 | | -113.1 | | |  |  |
| 0.7132 | 0.96726 | 1444 | -2.995 | 495 | | -70.1 | | |  |  |
| 0.8069 | 0.97546 | 1495 | -2.276 | 459 | | -27.9 | | |  |  |
| 0.9117 | 0.98224 | 1565 | -1.215 | 415 | | 16.9 | | |  |  |
| 0.9775 | 0.98514 | 1650 | -0.359 | 372 | | 29.4 | | |  |  |
| 1.0000 | 0.98576 | 1769 | 0 | 342 | | 0 | | |  |  |
| TBAH with 1-butanol at 293.15 K | | | | | | | | | |  |
| 0.0000 | 0.80980 | 1257 | 0 | 782 | | 0 | | |  |  |
| 0.0329 | 0.83801 | 1281 | -1.306 | 732 | | -34.3 | | |  |  |
| 0.0847 | 0.86776 | 1314 | -2.185 | 672 | | -68.8 | | |  |  |
| 0.1349 | 0.89302 | 1347 | -3.088 | 624 | | -93.4 | | |  |  |
| 0.1983 | 0.91730 | 1396 | -3.816 | 566 | | -121.2 | | |  |  |
| 0.2920 | 0.94551 | 1471 | -4.722 | 495 | | -147.6 | | |  |  |
| 0.3560 | 0.96081 | 1527 | -5.235 | 452 | | -159.7 | | |  |  |
| 0.4744 | 0.98169 | 1632 | -5.779 | 387 | | -168.0 | | |  |  |
| 0.5423 | 0.98989 | 1684 | -5.785 | 360 | | -162.5 | | |  |  |
| 0.5970 | 0.99507 | 1718 | -5.661 | 344 | | -152.8 | | |  |  |
| 0.6654 | 0.99997 | 1751 | -5.332 | 329 | | -135.4 | | |  |  |
| 0.7925 | 1.00574 | 1785 | -4.288 | 314 | | -89.4 | | |  |  |
| 0.9156 | 1.00739 | 1802 | -2.582 | 307 | | -38.2 | | |  |  |
| 0.9683 | 1.00707 | 1810 | -1.616 | 304 | | -16.1 | | |  |  |
| 1.0000 | 1.00285 | 1809 | 0 | 305 | | 0 | | |  |  |
| TBAH with 1-butanol at 298.15 K | | | | | | | | |  |  |
| 0.0000 | 0.80567 | 1240 | 0 | 807 | | 0 | | |  |  |
| 0.0329 | 0.83176 | 1260 | -1.120 | 761 | | -30.4 | | |  |  |
| 0.0847 | 0.86022 | 1284 | -1.911 | 708 | | -57.3 | | |  |  |
| 0.1349 | 0.88404 | 1319 | -2.693 | 654 | | -86.6 | | |  |  |
| 0.1983 | 0.90838 | 1362 | -3.484 | 596 | | -112.4 | | |  |  |
| 0.2920 | 0.93580 | 1434 | -4.351 | 522 | | -140.1 | | |  |  |
| 0.3560 | 0.95073 | 1482 | -4.852 | 481 | | -149.4 | | |  |  |
| 0.4744 | 0.97088 | 1580 | -5.333 | 414 | | -157.8 | | |  |  |
| 0.5423 | 0.97899 | 1630 | -5.345 | 386 | | -152.6 | | |  |  |
| 0.5970 | 0.98440 | 1660 | -5.278 | 370 | | -141.5 | | |  |  |
| 0.6654 | 0.98939 | 1694 | -4.978 | 353 | | -124.3 | | |  |  |
| 0.7925 | 0.99517 | 1728 | -3.965 | 337 | | -77.0 | | |  |  |
| 0.9156 | 0.99710 | 1753 | -2.334 | 327 | | -26.1 | | |  |  |
| 0.9683 | 0.99695 | 1772 | -1.413 | 320 | | -6.8 | | |  |  |
| 1.0000 | 0.99358 | 1798 | 0 | 311 | | 0 | | |  |  |
| TBAH with 1-butanol at 303.15 K | | | | | | | | |  |  |
| 0.0000 | 0.80195 | 1222 | 0 | 835 | | 0 | | |  |  |
| 0.0329 | 0.82612 | 1233 | -0.890 | 796 | | -22.3 | | |  |  |
| 0.0847 | 0.85456 | 1257 | -1.653 | 740 | | -51.2 | | |  |  |
| 0.1349 | 0.87813 | 1284 | -2.373 | 690 | | -75.1 | | |  |  |
| 0.1983 | 0.90195 | 1328 | -3.054 | 630 | | -102.4 | | |  |  |
| 0.2920 | 0.93003 | 1397 | -3.948 | 551 | | -132.4 | | |  |  |
| 0.3560 | 0.94506 | 1442 | -4.418 | 509 | | -141.2 | | |  |  |
| 0.4744 | 0.96576 | 1541 | -4.906 | 436 | | -152.8 | | |  |  |
| 0.5423 | 0.97443 | 1582 | -4.975 | 410 | | -143.4 | | |  |  |
| 0.5970 | 0.97992 | 1615 | -4.887 | 391 | | -133.8 | | |  |  |
| 0.6654 | 0.98524 | 1652 | -4.610 | 372 | | -117.6 | | |  |  |
| 0.7925 | 0.99172 | 1682 | -3.674 | 356 | | -66.9 | | |  |  |
| 0.9156 | 0.99379 | 1715 | -2.008 | 342 | | -17.2 | | |  |  |
| 0.9683 | 0.9936 | 1741 | -1.050 | 332 | | -0.1 | | |  |  |
| 1.0000 | 0.9917 | 1788 | 0 | 315 | | 0 | | |  |  |
| TBAH with 1-butanol at 308.15 K | | | | | | | | |  |  |
| 0.0000 | 0.79821 | 1207 | 0 | 860 | | 0 | | |  |  |
| 0.0329 | 0.82069 | 1212 | -0.684 | 826 | | -16.5 | | |  |  |
| 0.0847 | 0.84875 | 1234 | -1.376 | 769 | | -45.1 | | |  |  |
| 0.1349 | 0.87167 | 1257 | -1.985 | 721 | | -66.4 | | |  |  |
| 0.1983 | 0.89621 | 1297 | -2.723 | 658 | | -94.5 | | |  |  |
| 0.2920 | 0.92476 | 1361 | -3.628 | 580 | | -122.4 | | |  |  |
| 0.3560 | 0.93953 | 1404 | -4.020 | 536 | | -131.3 | | |  |  |
| 0.4744 | 0.96113 | 1505 | -4.587 | 457 | | -146.9 | | |  |  |
| 0.5423 | 0.97014 | 1543 | -4.678 | 431 | | -136.1 | | |  |  |
| 0.5970 | 0.97609 | 1575 | -4.650 | 412 | | -125.8 | | |  |  |
| 0.6654 | 0.98162 | 1614 | -4.381 | 389 | | -111.1 | | |  |  |
| 0.7925 | 0.98828 | 1642 | -3.423 | 374 | | -57.6 | | |  |  |
| 0.9156 | 0.99032 | 1681 | -1.689 | 356 | | -9.5 | | |  |  |
| 0.9683 | 0.99024 | 1708 | -0.730 | 345 | | 8.0 | | |  |  |
| 1.0000 | 0.98962 | 1777 | 0 | 320 | | 0 | | |  |  |
| TBAH with 1-butanol at 313.15 K | | | | | | | | |  |  |
| 0.0000 | 0.7942 | 1190 | 0 | 889 | | 0 | | |  |  |
| 0.0329 | 0.81608 | 1188 | -0.606 | 862 | | -9.0 | | |  |  |
| 0.0847 | 0.84448 | 1208 | -1.321 | 804 | | -37.1 | | |  |  |
| 0.1349 | 0.86701 | 1229 | -1.860 | 755 | | -58.2 | | |  |  |
| 0.1983 | 0.89039 | 1268 | -2.409 | 690 | | -86.9 | | |  |  |
| 0.2920 | 0.91701 | 1326 | -2.964 | 612 | | -112.4 | | |  |  |
| 0.3560 | 0.93132 | 1371 | -3.221 | 563 | | -124.8 | | |  |  |
| 0.4744 | 0.95215 | 1465 | -3.528 | 483 | | -138.5 | | |  |  |
| 0.5423 | 0.96146 | 1504 | -3.592 | 454 | | -128.6 | | |  |  |
| 0.5970 | 0.96803 | 1531 | -3.621 | 436 | | -116.2 | | |  |  |
| 0.6654 | 0.97488 | 1569 | -3.546 | 413 | | -100.7 | | |  |  |
| 0.7925 | 0.98339 | 1607 | -2.882 | 391 | | -50.4 | | |  |  |
| 0.9156 | 0.98703 | 1650 | -1.457 | 370 | | -1.9 | | |  |  |
| 0.9683 | 0.98686 | 1677 | -0.445 | 359 | | 16.4 | | |  |  |
| 1.0000 | 0.98737 | 1767 | 0 | 324 | | 0 | | |  |  |
| TBAH with 2-butanol at 293.15 K | | | | | | | | |  |  |
| 0.0000 | 0.80709 | 1229 | 0 | 820 | | 0 | | |  |  |
| 0.0850 | 0.85213 | 1271 | -0.553 | 726 | | -50.5 | | |  |  |
| 0.1318 | 0.87255 | 1300 | -0.891 | 677 | | -74.9 | | |  |  |
| 0.1990 | 0.89732 | 1343 | -1.345 | 618 | | -99.9 | | |  |  |
| 0.2862 | 0.92277 | 1399 | -1.794 | 553 | | -119.7 | | |  |  |
| 0.3570 | 0.93947 | 1460 | -2.093 | 499 | | -137.0 | | |  |  |
| 0.4589 | 0.95863 | 1542 | -2.381 | 439 | | -145.1 | | |  |  |
| 0.5990 | 0.97773 | 1617 | -2.446 | 391 | | -120.4 | | |  |  |
| 0.6936 | 0.98709 | 1666 | -2.267 | 365 | | -97.9 | | |  |  |
| 0.8232 | 0.99606 | 1709 | -1.628 | 344 | | -52.1 | | |  |  |
| 0.9249 | 1.00069 | 1764 | -0.818 | 321 | | -22.2 | | |  |  |
| 0.9714 | 1.00219 | 1782 | -0.345 | 314 | | -5.4 | | |  |  |
| 1.0000 | 1.00285 | 1809 | 0 | 305 | | 0 | | |  |  |
| TBAH with 2-butanol at 298.15 K | | | | | | | | |  |  |
| 0.0000 | 0.80267 | 1211 | 0 | 850 | | 0 | | |  |  |
| 0.0850 | 0.82988 | 1254 | 1.606 | 766 | | -37.6 | | |  |  |
| 0.1318 | 0.84622 | 1282 | 1.865 | 719 | | -59.3 | | |  |  |
| 0.1990 | 0.86867 | 1321 | 1.849 | 660 | | -82.7 | | |  |  |
| 0.2862 | 0.89526 | 1382 | 1.387 | 585 | | -110.3 | | |  |  |
| 0.3570 | 0.91376 | 1433 | 0.920 | 533 | | -124.4 | | |  |  |
| 0.4589 | 0.93546 | 1508 | 0.349 | 470 | | -132.5 | | |  |  |
| 0.5990 | 0.95553 | 1587 | 0.301 | 415 | | -111.6 | | |  |  |
| 0.6936 | 0.96465 | 1630 | 0.677 | 390 | | -86.1 | | |  |  |
| 0.8232 | 0.97483 | 1686 | 1.250 | 361 | | -45.6 | | |  |  |
| 0.9249 | 0.98414 | 1731 | 1.037 | 339 | | -12.8 | | |  |  |
| 0.9714 | 0.98945 | 1760 | 0.560 | 326 | | -0.7 | | |  |  |
| 1.0000 | 0.99358 | 1798 | 0 | 311 | | 0 | | |  |  |
| TBAH with 2-butanol at 303.15 K | | | | | | | | |  |  |
| 0.0000 | 0.79876 | 1193 | 0 | 880 | | 0 | | |  |  |
| 0.0850 | 0.82589 | 1230 | 1.673 | 800 | | -31.5 | | |  |  |
| 0.1318 | 0.84204 | 1250 | 1.985 | 759 | | -46.3 | | |  |  |
| 0.1990 | 0.86456 | 1294 | 1.996 | 690 | | -77.5 | | |  |  |
| 0.2862 | 0.89122 | 1345 | 1.566 | 620 | | -98.6 | | |  |  |
| 0.3570 | 0.91019 | 1389 | 1.055 | 569 | | -109.2 | | |  |  |
| 0.4589 | 0.9319 | 1458 | 0.528 | 505 | | -115.9 | | |  |  |
| 0.5990 | 0.95214 | 1550 | 0.513 | 437 | | -104.7 | | |  |  |
| 0.6936 | 0.96124 | 1598 | 0.941 | 407 | | -81.2 | | |  |  |
| 0.8232 | 0.97173 | 1656 | 1.503 | 375 | | -40.3 | | |  |  |
| 0.9249 | 0.98153 | 1705 | 1.207 | 350 | | -7.4 | | |  |  |
| 0.9714 | 0.98744 | 1732 | 0.589 | 337 | | 5.9 | | |  |  |
| 1.0000 | 0.99170 | 1788 | 0 | 315 | | 0 | | |  |  |
| TBAH with 2-butanol at 308.15 K | | | | | | | | |  |  |
| 0.0000 | 0.79446 | 1175 | 0 | 912 | | 0 | | |  |  |
| 0.0850 | 0.82150 | 1203 | 1.749 | 841 | | -20.3 | | |  |  |
| 0.1318 | 0.83773 | 1224 | 2.079 | 796 | | -37.7 | | |  |  |
| 0.1990 | 0.86036 | 1265 | 2.111 | 726 | | -68.2 | | |  |  |
| 0.2862 | 0.88746 | 1313 | 1.656 | 653 | | -89.1 | | |  |  |
| 0.3570 | 0.90636 | 1356 | 1.188 | 599 | | -101.2 | | |  |  |
| 0.4589 | 0.92825 | 1420 | 0.674 | 534 | | -106.0 | | |  |  |
| 0.5990 | 0.94872 | 1513 | 0.684 | 460 | | -97.2 | | |  |  |
| 0.6936 | 0.95790 | 1560 | 1.141 | 429 | | -72.6 | | |  |  |
| 0.8232 | 0.96856 | 1619 | 1.726 | 394 | | -30.9 | | |  |  |
| 0.9249 | 0.97887 | 1673 | 1.340 | 365 | | 0.1 | | |  |  |
| 0.9714 | 0.98505 | 1708 | 0.665 | 348 | | 11.0 | | |  |  |
| 1.0000 | 0.98962 | 1777 | 0 | 320 | | 0 | | |  |  |
| TBAH with 2-butanol at 313.15 K | | | | | | | | |  |  |
| 0.0000 | 0.79007 | 1157 | 0 | 946 | | 0 | | |  |  |
| 0.0850 | 0.81714 | 1180 | 1.806 | 878 | | -14.9 | | |  |  |
| 0.1318 | 0.83341 | 1202 | 2.159 | 830 | | -33.5 | | |  |  |
| 0.1990 | 0.85631 | 1240 | 2.187 | 759 | | -62.9 | | |  |  |
| 0.2862 | 0.88331 | 1285 | 1.787 | 685 | | -82.4 | | |  |  |
| 0.3570 | 0.90256 | 1322 | 1.293 | 633 | | -90.4 | | |  |  |
| 0.4589 | 0.92461 | 1381 | 0.794 | 566 | | -94.1 | | |  |  |
| 0.5990 | 0.94515 | 1472 | 0.855 | 488 | | -85.6 | | |  |  |
| 0.6936 | 0.95446 | 1524 | 1.331 | 451 | | -63.7 | | |  |  |
| 0.8232 | 0.96533 | 1585 | 1.925 | 412 | | -22.3 | | |  |  |
| 0.9249 | 0.97613 | 1647 | 1.454 | 377 | | 6.3 | | |  |  |
| 0.9714 | 0.98245 | 1678 | 0.751 | 361 | | 19.1 | | |  |  |
| 1.0000 | 0.98737 | 1767 | 0 | 324 | | 0 | | |  |  |
| TBAH with 2-methyl-2-propanol at 293.15 K | | | | | | | | |  |  |
| 0.0000 | 0.78576 | 1145 | 0 | 971 | | 0 | | |  |  |
| 0.0329 | 0.82926 | 1182 | -3.005 | 862 | | -86.4 | | |  |  |
| 0.0667 | 0.85547 | 1206 | -4.203 | 803 | | -123.1 | | |  |  |
| 0.0846 | 0.86986 | 1215 | -5.003 | 778 | | -136.4 | | |  |  |
| 0.1535 | 0.90969 | 1281 | -6.813 | 670 | | -198.8 | | |  |  |
| 0.1980 | 0.92852 | 1309 | -7.532 | 628 | | -210.9 | | |  |  |
| 0.2831 | 0.95365 | 1358 | -8.127 | 568 | | -214.2 | | |  |  |
| 0.3660 | 0.96819 | 1399 | -7.880 | 527 | | -199.7 | | |  |  |
| 0.5051 | 0.98318 | 1459 | -6.760 | 478 | | -156.5 | | |  |  |
| 0.6010 | 0.99046 | 1500 | -5.834 | 449 | | -121.6 | | |  |  |
| 0.7367 | 0.99908 | 1556 | -4.584 | 413 | | -66.9 | | |  |  |
| 0.8415 | 1.00347 | 1623 | -3.377 | 378 | | -32.1 | | |  |  |
| 0.9466 | 1.00524 | 1735 | -1.682 | 330 | | -9.9 | | |  |  |
| 1.0000 | 1.00285 | 1809 | 0 | 305 | | 0 | | |  |  |
| TBAH with 2-methyl-2-propanol at 298.15 K | | | | | | | | |  |  |
| 0.0000 | 0.78080 | 1123 | 0 | 102 | | 0 | | |  |  |
| 0.0329 | 0.81940 | 1140 | -2.499 | 938 | | -54.1 | | |  |  |
| 0.0667 | 0.84538 | 1154 | -3.715 | 887 | | -81.6 | | |  |  |
| 0.0846 | 0.85899 | 1178 | -4.450 | 838 | | -118.2 | | |  |  |
| 0.1535 | 0.89798 | 1240 | -6.220 | 724 | | -183.2 | | |  |  |
| 0.1980 | 0.91642 | 1271 | -6.915 | 675 | | -200.7 | | |  |  |
| 0.2831 | 0.94177 | 1315 | -7.565 | 614 | | -202.2 | | |  |  |
| 0.3660 | 0.95722 | 1358 | -7.462 | 566 | | -191.4 | | |  |  |
| 0.5051 | 0.97230 | 1409 | -6.367 | 518 | | -141.7 | | |  |  |
| 0.6010 | 0.97902 | 1452 | -5.335 | 485 | | -107.6 | | |  |  |
| 0.7367 | 0.98651 | 1515 | -3.834 | 442 | | -55.1 | | |  |  |
| 0.8415 | 0.99093 | 1588 | -2.602 | 400 | | -22.9 | | |  |  |
| 0.9466 | 0.99443 | 1702 | -1.301 | 347 | | -2.0 | | |  |  |
| 1.0000 | 0.99358 | 1798 | 0 | 311 | | 0 | | |  |  |
| TBAH with 2-methyl-2-propanol at 303.15 K | | | | | | | | |  |  |
| 0.0000 | 0.77531 | 1102 | 0 | 106 | | 0 | | |  |  |
| 0.0329 | 0.81085 | 1114 | -2.133 | 994 | | -43.8 | | |  |  |
| 0.0667 | 0.83659 | 1124 | -3.302 | 945 | | -67.4 | | |  |  |
| 0.0846 | 0.85058 | 1146 | -4.073 | 895 | | -104.3 | | |  |  |
| 0.1535 | 0.88910 | 1201 | -5.724 | 779 | | -168.2 | | |  |  |
| 0.1980 | 0.90777 | 1230 | -6.400 | 727 | | -186.7 | | |  |  |
| 0.2831 | 0.93431 | 1277 | -7.114 | 656 | | -194.6 | | |  |  |
| 0.3660 | 0.95128 | 1316 | -7.144 | 607 | | -182.2 | | |  |  |
| 0.5051 | 0.96759 | 1365 | -6.109 | 554 | | -130.6 | | |  |  |
| 0.6010 | 0.97426 | 1405 | -4.975 | 520 | | -93.4 | | |  |  |
| 0.7367 | 0.98155 | 1472 | -3.297 | 470 | | -42.1 | | |  |  |
| 0.8415 | 0.98630 | 1556 | -2.036 | 419 | | -15.0 | | |  |  |
| 0.9466 | 0.99126 | 1673 | -1.002 | 361 | | 5.2 | | |  |  |
| 1.0000 | 0.99170 | 1788 | 0 | 315 | | 0 | | |  |  |
| TBAH with 2-methyl-2-propanol at 308.15 K | | | | | | | | |  |  |
| 0.0000 | 0.76481 | 1080 | 0 | 112 | | 0 | | |  |  |
| 0.0329 | 0.79581 | 1087 | -1.551 | 106 | | -31.8 | | |  |  |
| 0.0667 | 0.82257 | 1096 | -2.802 | 101 | | -55.5 | | |  |  |
| 0.0846 | 0.83633 | 1115 | -3.523 | 961 | | -92.4 | | |  |  |
| 0.1535 | 0.87631 | 1160 | -5.236 | 848 | | -150.5 | | |  |  |
| 0.1980 | 0.89663 | 1187 | -6.045 | 791 | | -171.8 | | |  |  |
| 0.2831 | 0.92500 | 1232 | -6.848 | 712 | | -182.5 | | |  |  |
| 0.3660 | 0.94331 | 1269 | -6.918 | 658 | | -170.3 | | |  |  |
| 0.5051 | 0.96065 | 1318 | -5.786 | 599 | | -117.9 | | |  |  |
| 0.6010 | 0.96727 | 1357 | -4.454 | 561 | | -78.3 | | |  |  |
| 0.7367 | 0.97389 | 1433 | -2.359 | 500 | | -30.9 | | |  |  |
| 0.8415 | 0.98117 | 1507 | -1.486 | 449 | | 1.6 | | |  |  |
| 0.9466 | 0.98805 | 1630 | -0.775 | 381 | | 17.9 | | |  |  |
| 1.0000 | 0.98962 | 1777 | 0 | 320 | | 0 | | |  |  |
| TBAH with 2-methyl-2-propanol at 313.15 K | | | | | | | | |  |  |
| 0.0000 | 0.76507 | 1059 | 0 | 117 | | 0 | | |  |  |
| 0.0329 | 0.78907 | 1065 | -0.677 | 112 | | -21.0 | | |  |  |
| 0.0667 | 0.81000 | 1075 | -1.179 | 107 | | -41.4 | | |  |  |
| 0.0846 | 0.82021 | 1096 | -1.433 | 101 | | -80.1 | | |  |  |
| 0.1535 | 0.85296 | 1137 | -2.096 | 907 | | -129.8 | | |  |  |
| 0.1980 | 0.87081 | 1169 | -2.472 | 840 | | -159.4 | | |  |  |
| 0.2831 | 0.89817 | 1202 | -2.906 | 770 | | -157.7 | | |  |  |
| 0.3660 | 0.91886 | 1245 | -3.111 | 701 | | -156.2 | | |  |  |
| 0.5051 | 0.94399 | 1294 | -2.990 | 632 | | -108.9 | | |  |  |
| 0.6010 | 0.95645 | 1331 | -2.631 | 590 | | -69.8 | | |  |  |
| 0.7367 | 0.96979 | 1398 | -1.876 | 527 | | -18.4 | | |  |  |
| 0.8415 | 0.97772 | 1471 | -1.156 | 472 | | 14.6 | | |  |  |
| 0.9466 | 0.98441 | 1599 | -0.406 | 397 | | 27.6 | | |  |  |
| 1.0000 | 0.98737 | 1767 | 0 | 117 | | 0 | | |  |  |

**Table S2.** Estimated Parameters of eq 1 and Standard Deviation σ, for the Systems of ILs with butanol isomers as Function of Temperature.

| Y | System | T/K | a_0_ | a_1_ | a_2_ | σ |
| --- | --- | --- | --- | --- | --- | --- |
| *V^E^*/cm^3^.mol^-1^ | TPAH + 1-butanol | 293.15 | -14.470 | -14.722 | -48.301 | 0.167 |
|  |  | 298.15 | -14.488 | -12.164 | -37.774 | 0.127 |
|  |  | 303.15 | --15.365 | -9.006 | -24.540 | 0.087 |
|  |  | 308.15 | -15.852 | -6.880 | -16.466 | 0.060 |
|  |  | 313.15 | -16.454 | -6.302 | -5.778 | 0.038 |
|  | TPAH + 2-butanol | 293.15 | -6.228 | -8.785 | -32.837 | 0.111 |
|  |  | 298.15 | -6.785 | -6.734 | -25.063 | 0.085 |
|  |  | 303.15 | -15.657 | -7.020 | 0.0109 | 0.123 |
|  |  | 308.15 | -8.3881 | -2.808 | -10.545 | 0.035 |
|  |  | 313.15 | -9.449 | -1.337 | -3.315 | 0.014 |
|  | TPAH + 2-methyl-2-propanol | 293.15 | -22.104 | -7.027 | -48.293 | 0.130 |
|  |  | 298.15 | -18.058 | -2.212 | -43.976 | 0.103 |
|  |  | 303.15 | -20.356 | -1.947 | -20.658 | 0.050 |
|  |  | 308.15 | -20.387 | 0.839 | -11.156 | 0.018 |
|  |  | 313.15 | -14.617 | 0.421 | -1.789 | 0.004 |
|  | TBAH + 1-butanol | 293.15 | -1.092 | 10.230 | -0.880 | 0.005 |
|  |  | 298.15 | -18.128 | -2.387 | -3.714 | 0.010 |
|  |  | 303.15 | -18.572 | -3.319 | -10.880 | 0.022 |
|  |  | 308.15 | -19.352 | -4.586 | -18.231 | 0.040 |
|  |  | 313.15 | -20.517 | -4.723 | -22.787 | 0.049 |
|  | TBAH + 2-butanol | 293.15 | -9.598 | -3.075 | 0.363 | 0.013 |
|  |  | 298.15 | 0.842 | -3.361 | 24.458 | 0.003 |
|  |  | 303.15 | 1.748 | -2.909 | 25.039 | 0.001 |
|  |  | 308.15 | 2.283 | -2.250 | 26.519 | 0.001 |
|  |  | 313.15 | 2.755 | -1.498 | 27.978 | 0.003 |
|  | TBAH + 2-methyl-2-propanol | 293.15 | -23.996 | 22.497 | -38.965 | 0.058 |
|  |  | 298.15 | -23.170 | 21.859 | -28.191 | 0.042 |
|  |  | 303.15 | -22.545 | 21.344 | -20.023 | 0.033 |
|  |  | 308.15 | -22.369 | 19.660 | -9.415 | 0.027 |
|  |  | 313.15 | 5.943 | 28.650 | -73.291 | 3.774 |
| Δ*κ_s_* /T.Pa^−1^ | TPAH + 1-butanol | 293.15 | -694.246 | -4.567 | -122.326 | 2.905 |
|  |  | 298.15 | -694.635 | 314.777 | -436.819 | 2.520 |
|  |  | 303.15 | -701.958 | 308.781 | -191.790 | 2.346 |
|  |  | 308.15 | -649.767 | 318.771 | 48.638 | 1.341 |
|  |  | 313.15 | -661.176 | 291.371 | 114.883 | 1.352 |
|  | TPAH + 2-butanol | 293.15 | -518.951 | 368.804 | -210.318 | 3.707 |
|  |  | 298.15 | -508.677 | 389.066 | -53.555 | 2.132 |
|  |  | 303.15 | -572.082 | 542.638 | 390.028 | 4.681 |
|  |  | 308.15 | -618.799 | 632.102 | 796.293 | 5.075 |
|  |  | 313.15 | -656.628 | 707.293 | 1138.874 | 7.129 |
|  | TPAH + 2-methyl-2-propanol | 293.15 | -891.458 | 756.841 | -401.395 | 9.260 |
|  |  | 298.15 | -925.786 | 650.589 | 159.958 | 3.423 |
|  |  | 303.15 | -921.322 | 689.144 | 519.914 | 8.392 |
|  |  | 308.15 | -950.339 | 712.970 | 858.246 | 12.942 |
|  |  | 313.15 | -883.678 | 816.435 | 1122.566 | 10.623 |
|  | TBAH + 1-butanol | 293.15 | -638.212 | 229.619 | -120.496 | 7.114 |
|  |  | 298.15 | -614.470 | 281.995 | 71.364 | 7.277 |
|  |  | 303.15 | -604.612 | 285.493 | 271.760 | 6.902 |
|  |  | 308.15 | -591.780 | 310.395 | 453.308 | 9.699 |
|  |  | 313.15 | -581.889 | 326.389 | 659.538 | 12.531 |
|  | TBAH + 2-butanol | 293.15 | -551.880 | 213.651 | 128.039 | 3.265 |
|  |  | 298.15 | -552.487 | 190.469 | 294.588 | 2.730 |
|  |  | 303.15 | -495.681 | 201.865 | 424.346 | 8.108 |
|  |  | 308.15 | -477.385 | 204.365 | 576.696 | 10.022 |
|  |  | 313.15 | -455.158 | 255.635 | 711.164 | 10.169 |
|  | TBAH + 2-methyl-2-propanol | 293.15 | 542.029 | 1020.538 | -854.250 | 4.213 |
|  |  | 298.15 | -607.976 | 842.574 | -253.448 | 3.090 |
|  |  | 303.15 | -592.9753 | 799.140 | -30.404 | 9.366 |
|  |  | 308.15 | -574.713 | 797.9276 | 249.260 | 8.182 |
|  |  | 313.15 | -549.573 | 764.032 | 490.091 | 7.955 |
